# Characterization of continuum robot arms under reinforcement learning and derived improvements

**DOI:** 10.3389/frobt.2022.895388

**Published:** 2022-09-01

**Authors:** Ryota Morimoto, Masahiro Ikeda, Ryuma Niiyama, Yasuo Kuniyoshi

**Affiliations:** Graduate School of Information Science and Technology, The University of Tokyo, Tokyo, Japan

**Keywords:** soft robotics, continuum robot arm, reinforcement learning, reaching, crank rotation, throwing, peg-in-hole

## Abstract

In robotics, soft continuum robot arms are a promising prospect owing to their redundancy and passivity; however, no comprehensive study exists that examines their characteristics compared to rigid manipulators. In this study, we examined the advantages of a continuum robot arm as compared to a typical and rigid seven-degree-of-freedom (7-DoF) robot manipulator in terms of performing various tasks through reinforcement learning. We conducted simulations for tasks with different characteristics that require control over position and force. Common tasks in robot manipulators, such as reaching, crank rotation, object throwing, and peg-in-hole were considered. The initial conditions of the robot and environment were randomized, aiming for evaluations including robustness. The results indicate that the continuum robot arm excels in the crank-rotation task, which is characterized by uncertainty in environmental conditions and cumulative rewards. However, the rigid robot arm learned better motions for the peg-in-hole task than the other tasks, which requires fine motion control of the end-effector. In the throwing task, the continuum robot arm scored well owing to its good handling of anisotropy. Moreover, we developed a reinforcement-learning method based on the comprehensive experimental results. The proposed method successfully improved the motion learning of a continuum robot arm by adding a technique to regulate the initial state of the robot. To the best of our knowledge, ours is the first reinforcement-learning experiment with multiple tasks on a single continuum robot arm and is the first report of a comparison between a single continuum robot arm and rigid manipulator on a wide range of tasks. This simulation study can make a significant contribution to the design of continuum arms and specification of their applications, and development of control and reinforcement learning methods.

## 1 Introduction

Robot manipulators are commonly used in various applications. Among them, hyper-redundant manipulators and continuum robot arms have potential applicability in a wide range of applications, such as utilizing the degrees of freedom of the body. However, controlling them is problematic because of the softness of the material and degrees of freedom; this makes their modeling difficult. Dynamics models using machine learning have been proposed in prior studies (George Thuruthel et al., 2017; Thuruthel et al., 2017). Moreover, learning behavioral strategies through reinforcement learning can reduce human intervention in modeling and creating motor commands. Accordingly, learning using reinforcement learning can potentially solve the problem of soft robot control by developing data-driven control methods (Bhagat et al., 2019). Several studies have been conducted on reaching tasks using reinforcement learning (You et al., 2017; Zhang et al., 2017; Chattopadhyay et al., 2018; Satheeshbabu et al., 2019; Fetchrobotics, 2020; Morimoto et al., 2021). However, the research on soft robots and continuum robot arms, especially with regard to reinforcement learning, depends on the continuum robot arms owned by each research group, and there is no investigation into comparison with conventional “rigid” robots. In addition, the range of tasks performed by soft robots through reinforcement learning is more limited than that by rigid robots (Satheeshbabu et al., 2019). Under these circumstances, it would be useful to increase the number of tasks performed by the continuum robot arms through reinforcement learning and to analyze the characteristics of the continuum robot arms by comparing them with rigid seven-degree-of-freedom (7-DoF) robot manipulators, which are conventional rigid robot arms. Furthermore, examining the reinforcement learning based on the results is crucial.

Therefore, in this study, we analyzed the mechanical characteristics of the continuum robot arms through reinforcement learning while performing multiple tasks and compared the results with those for a conventional rigid 7-DoF robot manipulator. Furthermore, we propose a reinforcement learning method for continuum robot arms based on the results. In this study, the following four reinforcement learning tasks were employed: reaching, crank rotation, peg-in-hole, and ball throwing. This approach enabled further investigation into learning and control, differently from conventional robots to control, soft robots and continuum robot arms in particular.

Here, there are two distinct cases in the context of soft robotics: the case of soft body material itself, and the case of stiffness of the actuator of a robot with rigid links, i.e., impedance control of a robot with joints (Laschi and Cianchetti, 2014). This study focused on the former.

## 2 Related works

### 2.1 Continuum robot arms

A continuum robot arm is one of the most typical soft robots with a body that is flexible or connected by joints, such as ball or hinge joints, similar to a snake or an elephant’s trunk. Unlike robots with rigid links, a continuum robot arm can be bent and stretched at any point (Walker, 2013). Additionally, research is being conducted on robots that are mechanically similar to snakes and caterpillars (Hirose and Yamada, 2009; Ishige et al., 2018, 2019; Liu et al., 2020), for application in medicine as endoscopes (Ikuta et al., 1988) and in disaster sites (Kumar Singh and Krishna, 2014).

### 2.2 Soft robot and modeling

Soft robots are less likely to harm people than rigid robots because of their relatively low stiffness, and they are easier to grasp because of their compliance and ability to deform and compress. However, controlling a soft robot is often more difficult than controlling a rigid robot, which is made of rigid materials unlike a soft robot composed of soft materials. For example, manipulators of soft robots have a problem of infinite degrees of freedom because of their elasticity (George Thuruthel et al., 2018). While most conventional bulky robots are directly commanded by the motor of each joint, soft robots are manipulated considering the nonlinear deformation and elasticity caused by a movement (Wang et al., 2021).

The modeling of soft robots is usually accomplished either by detailed analysis through simulation or by rigorously solving approximate models mathematically while tolerating some degree of nonlinearity. Some studies have combined these methods with machine-learning or deep-learning methods to improve their performance (Han et al., 2020). However, these methods have several limitation, such as the inapplicability of models based on Cosserat theory (Rucker and Webster, 2011) to complex robots (Coevoet et al., 2017).

While the soft robot kinematics model can be approximated by rigid robot models using links and joints, the dynamics and contact model involves many parameters, and parameter estimation is often difficult. Although some research exists on obtaining the kinematics and dynamics of continuum robot arms by means of compartmentalized constant strain models, piecewise constant curvature methods, and other methods (Hannan and Walker, 2003; Webster and Jones, 2010; Renda et al., 2014; Escande et al., 2015), an approach different from that for rigid robots is necessary to solve the control problems of soft robots. In addition, some actuators have hysteresis that cannot be neglected, such as pneumatic artificial muscles, and some prior studies have modeled them (Zhang et al., 2019).

Therefore, data-driven control methods are considered useful for soft robots because of modeling difficulty, and the application of reinforcement learning has been proposed (Bhagat et al., 2019). Data-driven methods are often based on machine learning and include sampling data by actually moving the robot and modeling it using machine learning (Bruder et al., 2019; Buchler et al., 2018; George Thuruthel et al., 2017; Giorelli et al., 2015; Lee et al., 2017; Rolf and Steil, 2014; Thuruthel et al., 2017); and learning directly implemented on the controller by moving the robot and using reinforcement learning (Chattopadhyay et al., 2018; Morimoto et al., 2021).

Implementing reinforcement learning to a robot controller may fail if a command with excessive force is given during the learning process. However, limiting high torque to prevent breakage directly causes a narrow search range, which may in turn cause problems that are particularly incompatible with dynamic motion. While failure due to a high torque is a major problem for rigid robots, the body of a soft robot can absorb the vibrations caused by the high torque, and the softness of its body can reduce the impact if its motion is close to the prescribed limits. Therefore, soft robots and reinforcement learning may be compatible (Büchler et al., 2020).

### 2.3 Continuum robot arms and reinforcement learning

Much of the research on reinforcement learning for continuum robot arms is aimed at performing specific tasks using independently developed robots.

Investigations on reinforcement learning for continuum robot arms using models include: research on reaching and tracking using model-based reinforcement learning methods (Huang et al., 2018; Thuruthel et al., 2019) using guided policy search (Levine and Koltun, 2013); research using genetic algorithm (Goharimanesh et al., 2020); and research using a model-free reinforcement learning algorithm that learns and internally uses a forward model (Centurelli et al., 2022). Model-based reinforcement learning is feasible to some extent for continuum robot arms, which can be modeled and are relatively simple in structure and materials used. However, if the robot moves in 3D space or has a large number of actuators, regardless of whether the model is created by humans or acquired by learning using data-driven methods, the differences between the real and simulation robots increase, and the learning is adversely affected (Morimoto et al., 2021). Therefore, for selecting a reinforcement learning method that can be applied to many continuum robot arms, a model-free reinforcement learning method is preferable in which the robot model is neither provided by a user nor the forward model acquired through learning.

There are many studies on reaching tasks using model-free reinforcement learning algorithms. They range from using continuum robot arms with one segment (Chattopadhyay et al., 2018; Satheeshbabu et al., 2019, 2020) to two (Yang et al., 2019), three (Zhang et al., 2017), and four (You et al., 2017) segments. There are also other studies that use multi-agent reinforcement learning in which each actuator of a multi-degree-of-freedom arm is considered as one agent (Ansari et al., 2018; Perrusquía et al., 2020; Ji et al., 2021). Furthermore, there are studies that use reinforcement learning for the reaching component of the hierarchical control of tasks involving interactions with the environment of a continuum robot arm (Jiang et al., 2021).

If focusing on the robot rather than the task, no study has been conducted on continuum robot arms that simultaneously considers the structure and characteristics of the robot. There are studies that do not use reinforcement learning but learn to model kinematics and design a robot’s shape (Xu et al., 2021), but such studies end up focusing on the movement of the specific continuum robot arm.

To summarize, existing reinforcement learning research on continuum robot arms is essentially limited to the robots owned by each research group. Furthermore, the tasks themselves are mostly limited to reaching and tracking. Although there are cases where loads are considered, there are no reports on general-purpose tasks.

## 3 Experimental setup

### 3.1 Basics of reinforcement learning

In this paper, the following notations are used for reinforcement learning.

The state space S and action space A are considered continuous spaces. The number of time-steps in one episode is *T*, and the time-step *t* at a given point is represented by the discrete value for *t* ∈ [0, *T*]. The observation at a discrete timestep *t* is denoted by **s**_*t*_, and the command value output according to the policy *π*(**a**_*t*_|**s**_*t*_) is denoted by **a**_*t*_.

An immediate reward is given to an agent from the environment according to the reward function *r*_*t*_ = *r* (**s**_*t*_, **a**_*t*_). The return $R_{t}=\sum_{k=0}^{\infty }\gamma^{k}r_{t+k}$ is the sum of the discounted rewards from a time-step *t* using the discount factor *γ* ∈ [0, 1). Furthermore, the cumulative rewards, representing the episode rewards in one episode, are denoted by $\sum_{t=0}^{T}r_{t}$ .

A Markov decision process (MDP) consists of (S,A,p,r) , where *p* is the state transition probability, and $p:S\times S\times A\to \left[0,\infty \right)$ is the probability density of the state $s_{t+1}\in S$ for the next timestep *t* + 1, given the state $s_{t}\in S$ of the current timestep *t* and action $a_{t}\in A$ .

### 3.2 Soft actor-critic

In this section, soft actor-critic (SAC) (Haarnoja et al., 2018), the model-free reinforcement learning method mainly used in this study, is described. SAC is an off-policy reinforcement learning method. Moreover, it is a maximum entropy reinforcement learning method, which aims to improve robustness by maximizing the entropy and improve sample efficiency by using an off-policy method.

As SAC is a maximum entropy reinforcement learning, and its objective function contains an entropy term, as showcased by Eq. 1.

$$\pi^{*}={\arg\;\max}_{\pi }\underset{t}{\sum }E_{\left(s_{t},a_{t}\right)∼\rho_{\pi }}\left[r\left(s_{t},a_{t}\right)+\alpha H\left(\pi \left(\cdot |s_{t}\right)\right)\right],$$ (1)

where H(π(⋅|s)) is the entropy term, and *α* is the temperature parameter that determines the ratio of the entropy term to the reward term and determines the stochastic degree of the policy in outputting the action.

In SAC, the soft Q-function *Q*_*θ*_(**s**_*t*_, **a**_*t*_) and policy *π*_*ϕ*_(**a**_*t*_|**s**_*t*_) are considered, parameterized by the parameters *θ* and *ϕ*. In addition, three objective functions are used in SAC.

The first objective function is given by Eq. 2, which is a parameter of the soft Q-function to minimize the soft bellman residual.

$$J_{Q}\left(\theta \right)=E_{\left(s_{t},a_{t}\right)∼D}\left[\frac{1}{2}{\left(Q_{\theta }\left(s_{t},a_{t}\right)-\left(r\left(s_{t},a_{t}\right)+\gamma E_{s_{t+1}∼p}\left[V_{\bar{\theta }}\left(s_{t+1}\right)\right]\right)\right)}^{2}\right],$$ (2)

where D is the replay buffer, $\bar{\theta }$ is a parameter of target Q-network, and

$$V_{\theta }\left(s_{t}\right)=E_{a_{t}∼\pi }\left[Q_{\theta }\left(s_{t},a_{t}\right)-\alpha \log\pi_{\phi }\left(a_{t}|s_{t}\right)\right]$$ (3)

is the parameterized soft state value function.

The second objective function is an expression for the policy.

$$\begin{array}{c} J_{\pi }\left(\phi \right)=E_{s_{t}∼D,\epsilon_{t}∼N}\left[\alpha \log\pi_{\phi }\left(a_{t}|s_{t}\right)-Q_{\theta }\left(s_{t},a_{t}\right)\right], \end{array}$$ (4)

where *ϵ*_*t*_ is a noise sampled from a fixed probability distribution N .

The third objective function is related to the temperature parameter, which is computed using the dual problem, and it is given by Eq. 5.

$$J\left(\alpha \right)=E_{a_{t}∼\pi_{t}}\left[-\alpha \log\pi_{t}\left(a_{t}|s_{t}\right)-\alpha H\right].$$ (5)

Notably, two Q-networks and two target Q-networks are used to prevent bias in the policy update by preventing overestimation of Q-values. The Q-network with the lower Q-value is used in the calculation.

### 3.3 Continuum robot arm

#### 3.3.1 Real robot of continuum robot arm

In this study, a pneumatic continuum robot arm that has the same configuration as that proposed by Yukisawa *et al.* (Yukisawa et al., 2018) was used. The robot consists of nine bellows actuators, which are bellows-type pneumatic artificial muscles, referred to as extensible pneumatic actuator with bellows (EPAB) (Yukisawa et al., 2017). The actuator is composed of a rubber tube for realizing high extensibility, a bellows-shaped tube covering the rubber tube for limiting the elongation of the rubber in the longitudinal direction, and parts for fixing the rubber tube and braided tube. Three actuators are connected in parallel to form one segment, the natural length of each segment is 23 cm, and three segments are connected in series to form a robot. A continuum robot arm such as this one using EPAB is a relatively standard configuration and is not remarkably different from a suspended one using other actuators. Unlike the McKibben-type artificial muscles, this robot stretches without shrinking by utilizing compressed air. Compressed air of 0.35 MPa is supplied from the air compressor, and the pressure is adjusted by the valve to change the internal pressure of the actuator. The compressed air is supplied via a 38 L air tank and four 550 ml tanks to avoid pressure drop. There are nine actuators, one valve for each actuator, and the internal pressure can be independently controlled. One end of the robot is fixed and the other suspended. The information that can be observed is the position and velocity of points between the segments and an endpoint using an OptiTrack motion capture system (Natural Point, Inc.), and the internal pressure of each rubber tube using pressure sensors. In addition, to verify if there is insufficient supply of compressed air, the internal pressure just before air is supplied to each valve is also observed.

The observation information of this robot has the following 36 dimensions: positions between segments (3 dimensions, 3 items), velocity of each point between segments (3 dimensions, 3 items), inner pressure of each EPAB (1 dimension, 9 items), and time derivative of inner pressure of each EPAB (1 dimension, 9 items). Nine valves are controllable and can be independently controlled.

#### 3.3.2 Simulation of continuum robot arm

**FIGURE 1:**
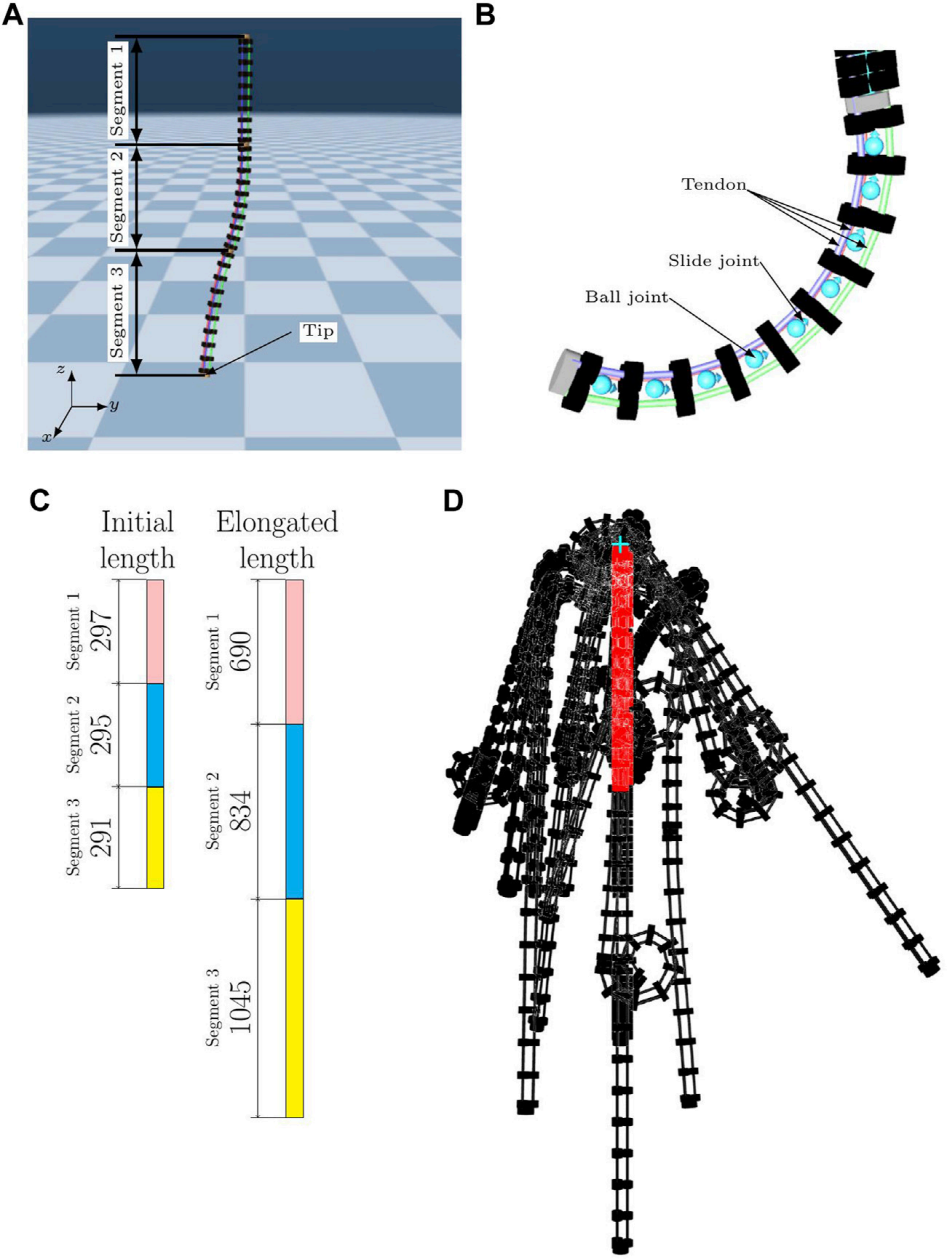
**(A)** Continuum robot arm model in the simulation. **(B)** Light blue objects represent the slide and ball joints. **(C)** Original length of each segment and the length of each segment when most elongated by the actuator. **(D)** Examples of the posture when the actuator is operated are shown in black. The red one is the original posture.

To simulate the robot, a robot model was developed using the Multi-Joint dynamics with Contact (MuJoCo) (Todorov et al., 2012) physics engine to mimic a real pneumatic continuum robot arm, as shown in Figure 1A (Morimoto et al., 2021). The actuators of the robot in this simulation are different because the real robot is pneumatically driven, whereas in the simulation, cylinders are used to drive the tendons, which are strings in MuJoCo. The body of the robot is composed of EPABs, which are artificial muscles in the real robot. However, each EPAB is replaced by 10 cylinders in the simulation, which play the roles of mass, inertia, and contact.

For the geometric constraint, this continuum robot arm has only one virtual body at the center of the three tendons in each segment, and the distance from the center of the body is constrained. Each cylinder has a fixed distance from the center and forms a shape by being completely fixed to the same cylinder that is attached to another tendon in the same segment and in the same order counting from the end, similar to a real robot. Moreover, each cylinder is fixed to a tendon to reproduce a single artificial muscle. Considering the actuators, the cylinders attached to each of the nine tendons can be independently controlled. Considering the degrees of freedom, the robot can extend and rotate by connecting three fixed cylinders with slide and ball joints, as shown in Figure 1B. The range of motion of the robot is also shown in Figures 1C,D. In the simulation, the robot is suspended in the air with its upper end fixed, similar to a real robot. Gravity applies to the robot in the simulation in the same way that it does in the real world.

The observation information of this robot has the following 36 dimensions: position of each point (3 dimensions, 3 items), velocity of each point (3 dimensions, 3 items), length of each tendon, which corresponds to the internal pressure of the EPAB in a real robot (1 dimension, 9 items), and time derivative of the length of each tendon (1 dimension, 9 items).

### 3.4 7-DoF arm robot for comparison

**FIGURE 2:**
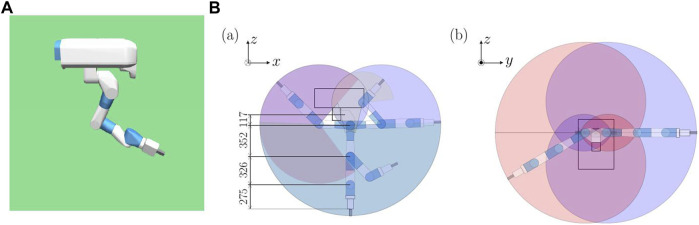
7-DoF arm robot hanging model. **(A)** The backward, leftward, and upward directions of the image are the positive x, y-, and z-axis directions respectively. **(B)** Range of motion of the robot.

In this section, the rigid 7-DoF robot manipulator is described, which is used for comparison to investigate the characteristics of continuum robot arms. In this study, the 7-DoF arm of Fetch robot (Fetch Robotics) (Wise et al., 2016) was used as the 7-DoF arm robot for comparison. The model of the continuum robot arm is such that its one end is fixed in space, and the model is suspended from it. To apply this condition to the 7-DoF arm robot, a model hanging from the original space is created based on the Fetch model (Figure 2A). This environment is created by extracting the 7-DoF arm robot and part of the body from the original environment, fixing them in the air, and hanging them. Gravity applies to the robot in the simulation in the same way that it does in the real world. It is not normal for a 7-DOF arm robot to be fixed in a dangling position. The 7-DoF arm robot can exert sufficient force to defy gravity. Therefore, when considering kinematics and workspace, the situation is not so different from that of an arm robot approaching a vertically placed crank or hole. Therefore, this study concluded that there is no change in the task’s difficulty when the 7-Dof arm robot performs the task in a dangling position. The range of motion of the robot is shown in Figure 2B.

The simulation environment for this robot is the model used in the Fetch environment of OpenAI/Gym (Brockman et al., 2016). To control the robot, position motors are installed at each joint and operated to move the robot. The parameters for the torque information and proportional gain are obtained from the parameters used in the manuals (Fetchrobotics, 2020) and mujoco-py (Ray et al., 2021).

For reinforcement learning, the joint angles and joint angular velocities of the seven joints are used as the observation data. For the controllable values, the position servo motors embedded in the joints are used. Moreover, if a gripper is used, the position servo motor for moving the gripper as well as the displacement and velocity of the gripper are added to the observation data.

### 3.5 Description of tasks and environment

In this section, the original reinforcement learning tasks used in this study and the environment for them are described. The list of tasks and each element to be compared is shown in Table 1.

**TABLE 1:** The list of tasks and elements to be compared.

Special characteristic	Crank rotation	Peg-in-hole	Boll thorowing
Redundancy	*✓*	*✓*	—
Anisotropy	—	—	*✓*
Precise control	—	*✓*	—
Dynamic movement	*✓*	—	*✓*
Influence of the initial state of the robot	*✓*	*✓*	(Not affected by initial state)
Impact of environmental clutter	*✓*	*✓*	—

#### 3.5.1 Reaching

Reaching is employed as a basic position-control task of the robot. Experiments for this task were performed only on the continuum robot arm.

In this environment, the objective was to get the robot’s endpoints closer to the target point as quickly as possible.

Experiments in this task were conducted both on the real robot and on the simulation model that imitates it. These experiments were performed to demonstrate that reinforcement-learning experiments in the simulation can be applied to the real robot.

In this environment, the position of the target point (3 dimensions) was added to the state space in reinforcement learning. The number of time-steps for each episode, *T*, was fixed at 300 steps and the episode did not terminate in the middle. The period of the simulation was 2 ms. Additionally, the same action was repeated 20 times, resulting in a policy time-step of 40 ms or a frequency of 25 Hz. In addition, 334 episodes were conducted for each experiment, that is, a total of approximately 100 k steps of data were collected.

The target point for reaching was randomly determined within a pre-determined area at the initialization of every episode. The area consists of a cylinder with a height of 30 cm and radius 30 cm, and a cone with a height of 30 cm above it. The center of the bounding circle is below the fixed point of the continuum robot arm. The bounding circle is horizontal to the ground.

The reward function *r*(*t*) is defined as

$$r\left(t\right)=-\|x\left(t\right)-g\left(t\right)\|,$$ (6)

where **x**(*t*) is the position of the tip of the manipulator at time-step *t*, and **g**(*t*) is the position of the target point. Notably, **x**(*t*) = (*x*_*x*_(*t*), *x*_*y*_(*t*), *x*_*z*_(*t*)), and **g**(*t*) = (*g*_*x*_(*t*) *g*_*y*_(*t*), *g*_*z*_(*t*)). Therefore, the close the manipulator tip is to the target point, the higher is the reward. Accordingly, if the tip of the manipulator approaches the target point as quickly as possible, the accumulated reward is large.

In addition, this reward function only takes values less than or equal to zero; therefore, the episode reward is negative. The position of the manipulator, other than the tip of the manipulator, is not relevant to the reward.

#### 3.5.2 Crank rotation

Crank rotation is employed as a robot task related to classical position control. In addition, an environment was created with an obstacle between the robot and crank to investigate the effect of the obstacle on learning.

**FIGURE 3:**
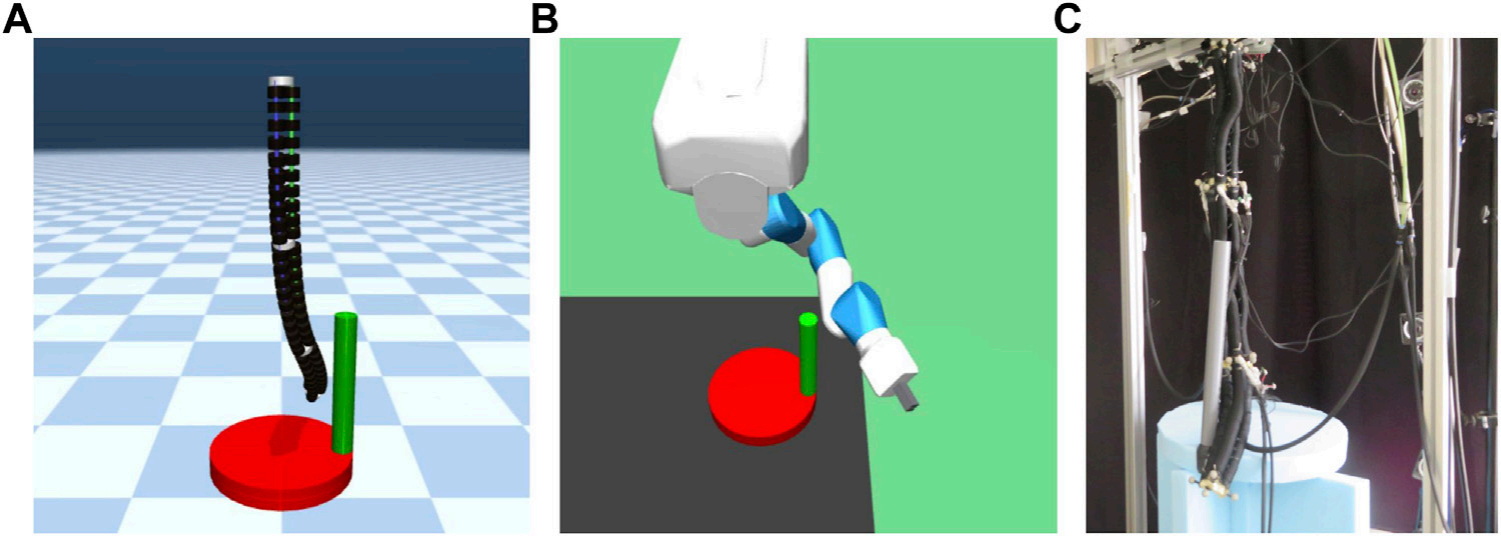
Crank model. **(A)** is continuum robot arm model, **(B)** is 7-DoF arm robot model, and **(C)** shows an actual continuum robot arm performing a crank rotation task.

This environment is an original environment as shown in Figure 3. In this environment, the goal is to turn the crank at the bottom of the robot as fast as possible in the same direction with the robot arm hanging in the air. As shown in Figure 3C, a real continuum robot arm can perform this task from a kinematic point of view.

##### 3.5.2.1 Setting up experiments in crank rotation task

The continuum robot arm is initially in the extended state and hanging downwards. In the continuum robot arm environment, the distance between the lowest point in the initial state and disk of the crank was 17.5 cm if there was no postural randomness as described below, and the distance between the crank and upper end, which is the fixed point of the continuum robot arm, was 1.0 m.

The initial state of the 7-DoF arm robot was set similar to the original initial condition set by OpenAI. In the 7-DoF arm robot environment, the distance between the fixed point and disk of the crank was 1.0 m if there was no postural randomness. As for the horizontal position relationship, the axis of rotation of the disk was almost directly below the lowest point of the initial state and almost directly below the position of the uppermost link for the continuum arm and 7-DoF arm robot, respectively.

The radius of the disk was 20 cm, and its height was 6 cm. The orientation of the disk was such that the axis of the cylinder was vertical to the ground. In addition, a handle was installed on the disk. The handle was 3 cm in diameter, 40 cm in length, and was fixed such that the axis of the handle’s cylinder existed at a point 17 cm from the center of the disk. The axis of the handle was fixed at a point 17 cm from the center of the disk and aligned vertically with the ground.

##### 3.5.2.2 Environment

In this environment, the position in 3-D space of the crank (3 dimensions), rotation angle (1 dimension), and rotation angular velocity (1 dimension) are added to the state space in reinforcement learning. Notably, the gripper was not used.

The number of time-steps, *T*, for each episode was fixed at 1000 and the episode did not terminate in the middle. The period of the simulation is 2 ms, and the same action is repeated 20 times, resulting in a policy time-step of 40 ms. In addition, 1000 episodes were conducted for each experiment.

The reward function *r*(*t*) at time-step *t* is defined as

$$r\left(t\right)=\dot{\theta }\left(t\right),$$ (7)

where $\dot{\theta }\left(t\right)$ is the angular velocity of the crank. A higher reward is given for faster rotation of the crank in a particular direction. There are no constraints to prevent the manipulator from moving away from the handle or disk and no rewards to encourage this.

In addition, a penalty term is not set for subtracting the reward if the addition to the actuator is large. This is because, as mentioned above, the need for penalty terms is smaller for soft robots than for rigid robots. Additionally, penalty terms are not introduced because they can inhibit learning.

#### 3.5.3 Peg-in-hole

Peg-in-hole is adopted as a task related to classical robot manipulation, which requires both position and force control. Furthermore, similar to the crank rotation environment, an environment was created with obstacles between the robot and crank to investigate the effect of obstacles on learning.

**FIGURE 4:**
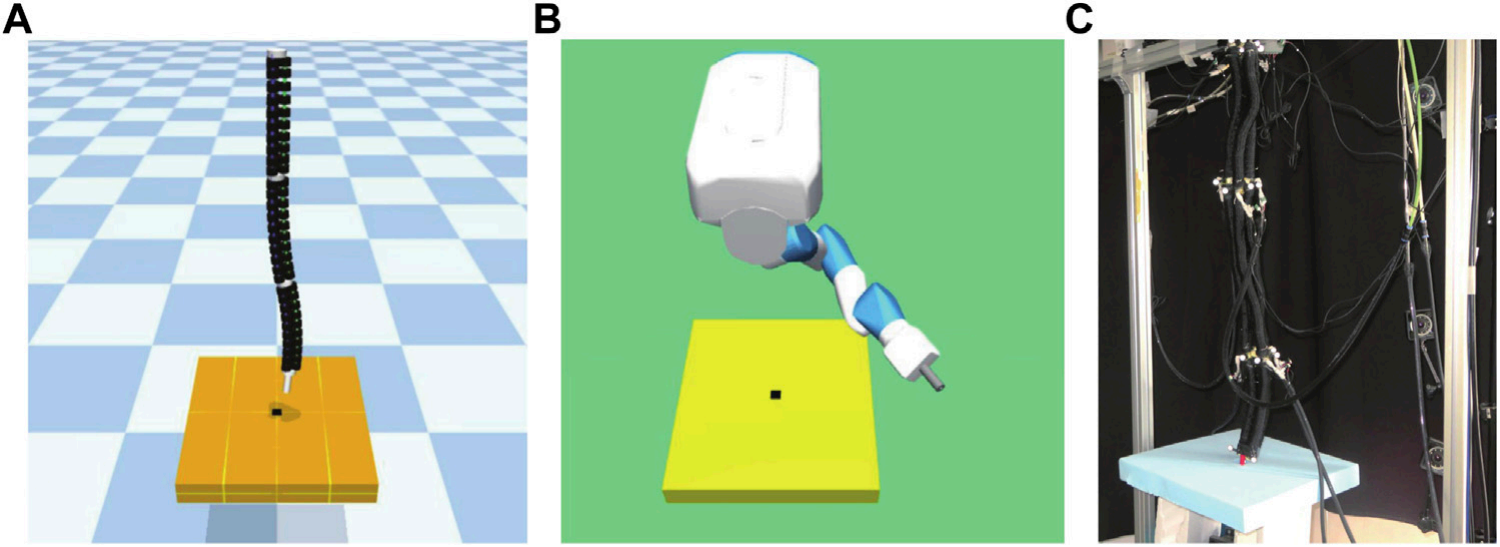
Peg-in-hole model. **(A)** is continuum robot arm model, **(B)** is a 7-DoF arm robot model. The rightward, backward, and upward directions of the board are the positive x-, y-, and z-axis directions, respectively, and **(C)** shows an actual continuum robot arm performing a peg-in-hole task.

This environment is an original environment as shown in Figure 4. In this environment, the objective was to insert a stick attached to the end of a robot arm hanging in the air into a hole at the bottom of the robot as quickly as possible. As shown in Figure 4C, a real continuum robot arm can perform this task from a kinematic point of view.

##### 3.5.3.1 Setting up experiments in peg-in-hole task

A rod with a radius of 1.5 cm and length 10 cm was attached to the end of the robot for both the continuum robot arm and 7-DoF arm robot. A square plate of 40 cm length square with a 4 cm length square hole was located underneath the robot. Its thickness was 10 cm, the same as the length of the rod.

The peg was fixed to the end of the continuum robot arm or the end-effector of the 7-DoF arm robot. The orientation of the peg was the same as the orientation of the tip of the continuum robot arm or the end-effector of the 7-DoF arm robot.

In the continuum robot arm environment, the distance between the plate and the lowest point of the robot was 17.5 cm. Additionally, the distance between the plate and upper end of the robot, which is the fixed point of the continuum robot arm, was 1.0 m. In the 7-DoF arm robot environment, the distance between the fixed point and board was 1.0 m if there was no postural randomness. Considering the relationship of the horizontal positions, the hole was almost directly under the lowest point of the initial condition and almost directly under the position of the uppermost link for the continuum robot arm and 7-DoF arm robot, respectively.

##### 3.5.3.2 Environment

In this environment, the position of the hole (3 dimensions) was added to the state space in reinforcement learning. The number of time-steps for each episode, *T*, was fixed at 1000 and the episode did not terminate in the middle. The period of the simulation was 2 ms. Additionally, the same action was repeated 20 times, resulting in a policy time-step of 40 ms or a frequency of 25 Hz. In addition, 1000 episodes were conducted for each experiment.

The reward function *r*(*t*) is defined as.

$$r\left(t\right)=-\|x\left(t\right)-g\left(t\right)\|+I\left(t\right),$$ (8)

$$\begin{array}{ll} \text{where}\\ I\left(t\right) & =\left\{\begin{array}{cc} 1, & |x_{x}\left(t\right)-g_{x}\left(t\right)|<r_{h}\cap |x_{y}\left(t\right)-g_{y}\left(t\right)|<r_{h}\cap |x_{z}\left(t\right)-g_{z}\left(t\right)|<0.1cm\\ 0, & \text{else} \end{array}\right., \end{array}$$

Where **x**(*t*) is the position of the tip of the rod attached to the manipulator at time-step *t*, **g**(*t*) is the position of the deepest part of the hole, and *r*_*h*_ is the length of one side of the square hole. Notably, **x**(*t*) = (*x*_*x*_(*t*), *x*_*y*_(*t*), *x*_*z*_(*t*)), **g**(*t*) = (*g*_*x*_(*t*) *g*_*y*_(*t*), *g*_*z*_(*t*)). The first term on the right-hand side of the reward function is the auxiliary reward such that the closer the stick is to the hole, the more the reward is given. The second term on the right-hand side is the success reward, which is given if the stick goes deep into the hole. Accordingly, if the stick approaches the hole as quickly as possible and goes deep into the hole, the accumulated reward is large. Similar to crank rotation, a penalty term is not set for this case that subtracts the reward if the addition to the actuator is large.

This setup results in the number of time-steps in one episode, *T*, being fixed at 1000; therefore, the maximum cumulative reward is 1000, and it never actually reaches 1000 because there is a negative reward for reaching as an auxiliary reward. In addition, although two types of robots were compared, the minimum time to reach the time-step *t* (where *I*(*t*) = 1) is considered different because the properties of the two robots are different; therefore, a comparison for the maximum value of the cumulative reward is not strictly possible.

#### 3.5.4 Ball throwing

In this experiment, we investigated the effect of the properties of continuum robot arms, such as direction dependence, on reinforcement learning by performing a throwing task that requires the robot to apply force to an object other than the robot.

**FIGURE 5:**
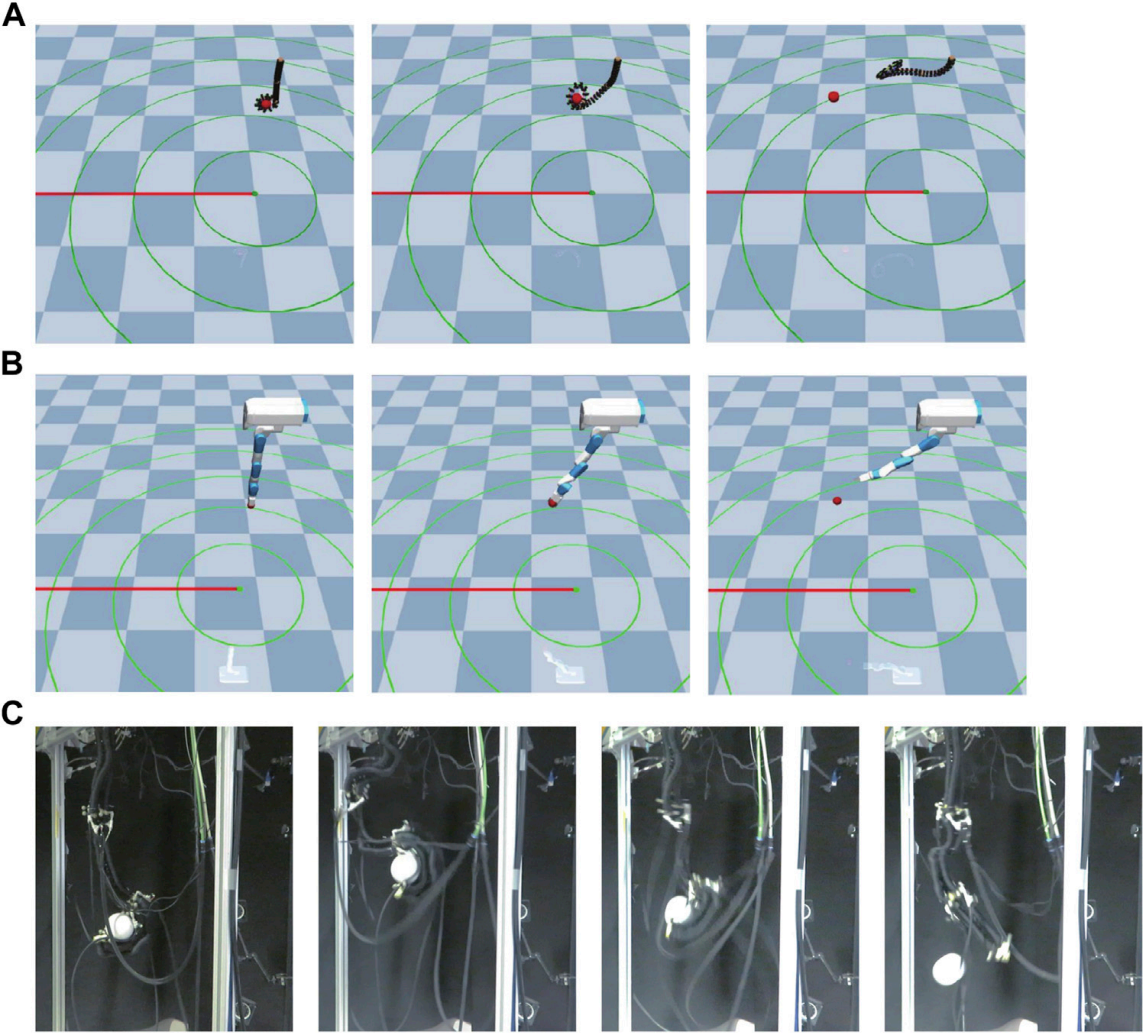
Ball throwing model. **(A)** is continuum robot arm model, **(B)** is 7-DoF arm robot model, and **(C)** shows an actual continuum robot arm performing a ball throwing task.

This environment is an original environment as shown in Figure 5. The goal of this environment is to throw a ball grasped at the end of a robot arm hanging in the air as far as possible in a specified direction.

##### 3.5.4.1 Setting up experiments in ball throwing task

The continuum robot arm grasps the ball at the start of an episode by bending the bottom segment, whereas the 7-DoF arm robot is manipulated by two plates (grippers) attached to the end of the robot that act as fingers and move independently by actuators. As in the other environments, the upper end of the robot was fixed and did not rotate; however, for the 7-DoF arm robot, we created another environment in which the root can rotate.

##### 3.5.4.2 Environment

In this environment, the position (3 dimensions) and velocity (3 dimensions) of the ball as well as the target direction vector (2 dimensions) of the throw on the plane were added to the state in reinforcement learning at a certain time *t*. For the 7-DoF arm robot, information about its position (1 dimension x 2) and velocity (1 dimension x 2) were added to the state space because a gripper grasps the ball. Moreover, an actuator was added to the action space for moving the gripper (1 dimension x 2). In addition, a 7-DoF arm robot environment with a rotatable root was created in this experiment to check the effect of symmetry. In this environment, the joint rotation angle (1 dimension) and rotation angular velocity (1 dimension) for root rotation were added to the state space. Moreover, the actuator (1 dimension) was added to the action space for moving the joint.

The number of time-steps for each episode, *T*, was fixed at 150, and does not end in the middle of the episode. The simulation period is 2 ms, and the same action is repeated 20 times, resulting in a policy time-step of 40 ms or a frequency of 25 Hz. In addition, 5000 episodes are conducted for each experiment.

The reward *r*(*t*) is defined as

$$r\left(t\right)=\left(b_{t}-b_{0}\right)\cdot t,$$ (9)

where **b**_*t*_ is the ball position at timestep *t*, **b**_0_ is the initial ball position, and **t** is the target direction vector from the initial ball position of the throw. Moreover, a higher immediate reward is obtained if the ball exists farther in the target direction.

### 3.5.5 Characteristics of each task

We believe that evaluating robots in the tasks focused on in this paper will allow us to investigate the properties required for arm robots. The Table 1 shows the characteristics of the robots to evaluate in each task.

We first focus on redundancy as a property of continuum robot arms. We can check the robot arm’s redundancy with crank rotation and peg-in-hole tasks. The ball-throwing task confirms the anisotropy of the robot. In addition, arm robot locomotion can be static and requires precision or dynamic movement; the peg-in-hole task evaluates precision control performance. The ball-throwing and crank-turning tasks assess dynamic locomotion. Crank rotations and peg-in-hole confirm the effects of environmental clutter. We believe that the task-focused in this study confirms many of the characteristics required for a continuum robot arm.

## 4 Comparison of real robot and simulation model

In this section, we confirm that the simulation model used in this study can play a role in replacing the real robot. For this purpose, reaching experiments were conducted with both the real robot and the simulation, and they were compared to confirm the validity of the model.

### 4.1 Methods

A reaching environment was used in this experiment. SAC was used as the reinforcement-learning method. The SAC parameters are the same as in (Haarnoja et al., 2018). The experiments with real robots used a single GeForce RTX 2080 Ti GPU and an Intel Octa-Core Processor i9-9900K CPU. The learning method and environmental setup are the same.

Only two experiments were conducted with real robots, and 30 experiments were conducted with simulations. In the experiment using the real robot, time must be allowed between episodes to fill the tank with compressed air and to suppress the swaying of the continuum robot arm. Furthermore, the rubber tube of the robot sometimes punctures, and its repair also requires time. Therefore, it took approximately 20 h to collect and train 100 k steps of data. Therefore, only two experiments were conducted using a real robot.

### 4.2 Result and discussion

**FIGURE 6:**
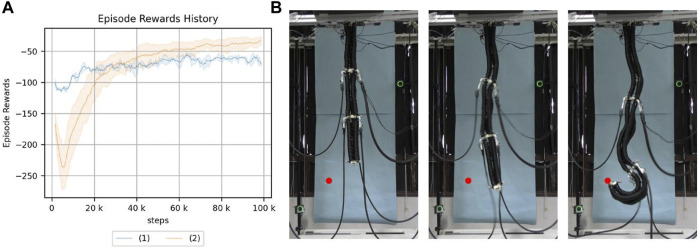
**(A)** Return of reaching. (1): the real robot, (2): the simulation model. **(B)** Reaching with a real robot using a learned policy. The red point is the target point.

The experimental results are shown in Figure 6.

The final results and learning progress are different because of the strictly different characteristics of the robots, but the trends are similar. The mean final cumulative reward is −76.4 for the real robot and −36.6 for the simulation.

This suggests that the simulation used in this study is useful as an experimental environment that can substitute for a real robot with a continuum robot arm. Therefore, subsequent experiments will be conducted using the simulation to improve the experimental efficiency.

## 5 Characteristic analysis of continuum robot arms using reinforcement learning

### 5.1 Methods

SAC was used as the reinforcement learning method. The SAC parameters were the same as in (Haarnoja et al., 2018).

#### 5.1.1 Setting up experiment to investigate effect of randomness on learning

This section details the effects of randomness in the initial state of the robot and environment on learning.

##### 5.1.1.1 Randomness of environment

For environmental randomness, different randomness criteria were considered for crank rotation and peg-in-hole.

For crank rotation, two randomness criteria were considered: whether the initial angle of the crank handle is randomly selected from a point on the circumference for each episode and whether the position of the crank changes for each episode. The axis of rotation of the crank did not change. If the initial angle of the handle was randomly determined, the initial state rotation angles of the disk and handle were determined by sampling from 360° using a uniform distribution. If the position of the crank changes for each episode, it was determined by uniformly sampling from the area of a cylinder with a radius of 20 cm and a height of 40 cm. The orientation of the cylinder was such that its axis was vertical to the ground.

Next, for the peg-in-hole, a random change was considered in the position of the board with a hole for each episode. The randomness was determined by uniformly sampling the area of a cylinder of radius *c* ∈ {2, 5, 10 cm} and height *c* [cm] centered on a reference point in the direction horizontal to the ground with constant *c*.

##### 5.1.1.2 Randomness of Robot’s state at initialization of environment

The effect of randomness in the robot’s initial pose was investigated to analyze the characteristics of a continuum robot arm using reinforcement learning.

The randomness of the robot’s initial state was determined by whether the initial angle and angular velocity of each joint in the robot are randomized at the beginning of the episode in reinforcement learning. For this randomness comparison, three different environments were created in the experiment: one without randomness, and the other two with different degrees of randomness. The degree of randomness was set differently for the continuum robot arm and the 7-DoF arm robot, and the number of joints in the robot was considered so that the degree of randomness is almost the same. To achieve this, the range of the random sampling was set 10 times different for the value used to determine the randomness, taking into account the difference in passive degrees of freedom.

For the continuum robot arm, the randomness of each joint was independently sampled from −0.001 to 0.001 for low randomness and from −0.01 to 0.01 for high randomness, using a uniform distribution. The values were added to the default values using the arc degree method for ball joints and centimeters for sliding joints. For the 7-DoF arm robot, the initial angle of each joint was randomly sampled using a uniform distribution independently from −0.01 to 0.01 for low randomness and from −0.1 to 0.1 for high randomness. The values were added to the default value using the arc degree method.

##### 5.1.1.3 Setting up experiment to investigate effect of obstacles on learning

**FIGURE 7:**
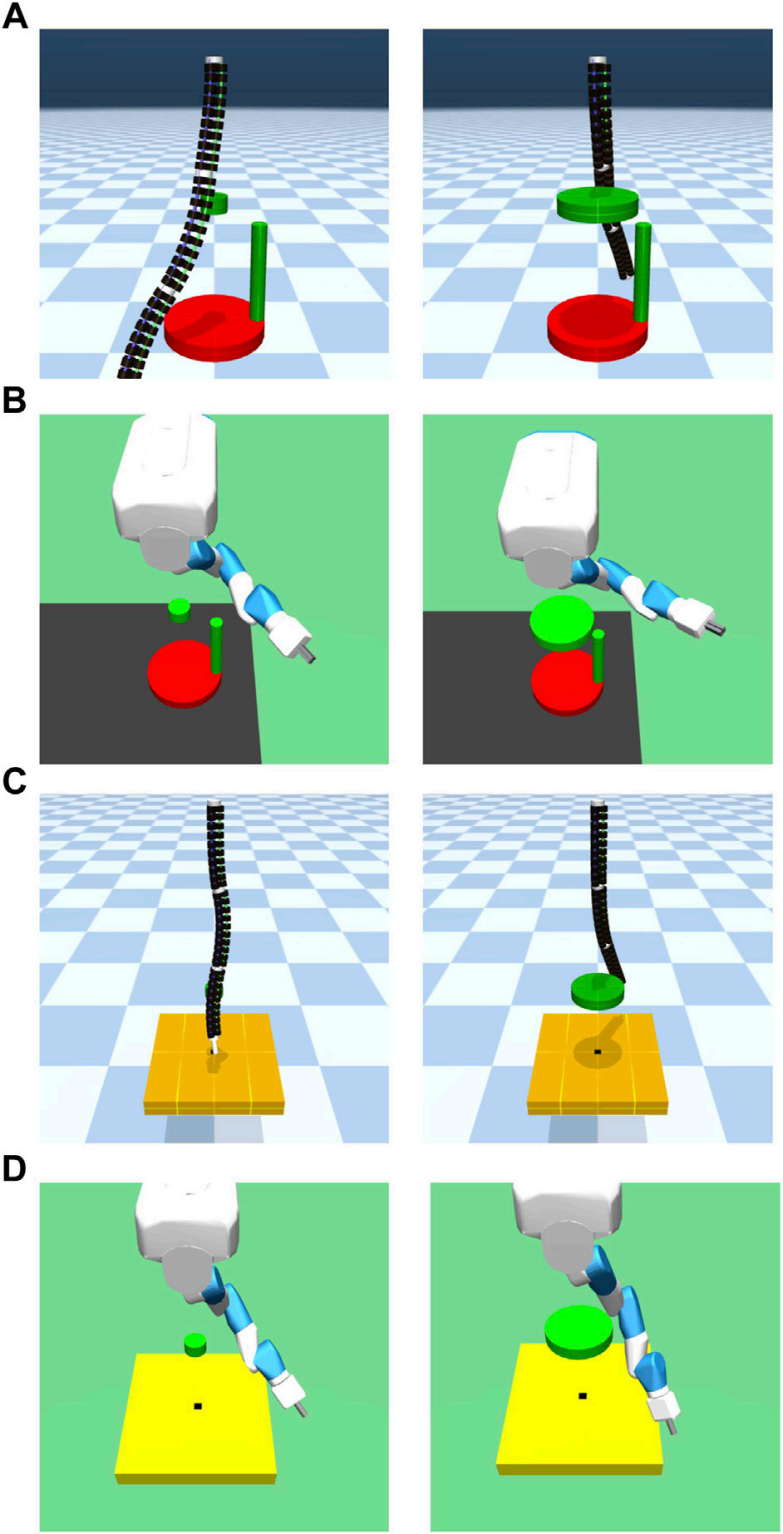
Environment with obstacle. **(A,B)** are crank models. **(C,D)** are peg-in-hole models. **(A,C)** are continuum robot arm models. **(B,D)** are 7-DoF arm robot models.

An environment was created to check the influence of obstacles by placing obstacles between the initial position of the robot manipulator and crank in the crank rotation environment as well as between the initial position of the robot manipulator and hole in the peg-in-hole environment. The situation is illustrated in Figure 7. As illustrated, obstacles were placed at fixed positions in the environment to observe their effects. The large obstacle had a radius of 15 cm and height 6 cm. The medium-sized obstacle had a radius of 10 cm and height 6 cm. The small obstacle had a radius of 5 cm and a height of 6 cm. Note that only crank rotation was set for the obstacles of intermediate size. The obstacle floated 50 cm above the crank and the board with the hole.

### 5.2 Result

In this section, the results of the experiments are presented. Note that, the same type of experiment was performed 16 times, each with different seed values.

#### 5.2.1 Results and discussion of crank rotation

**FIGURE 8:**
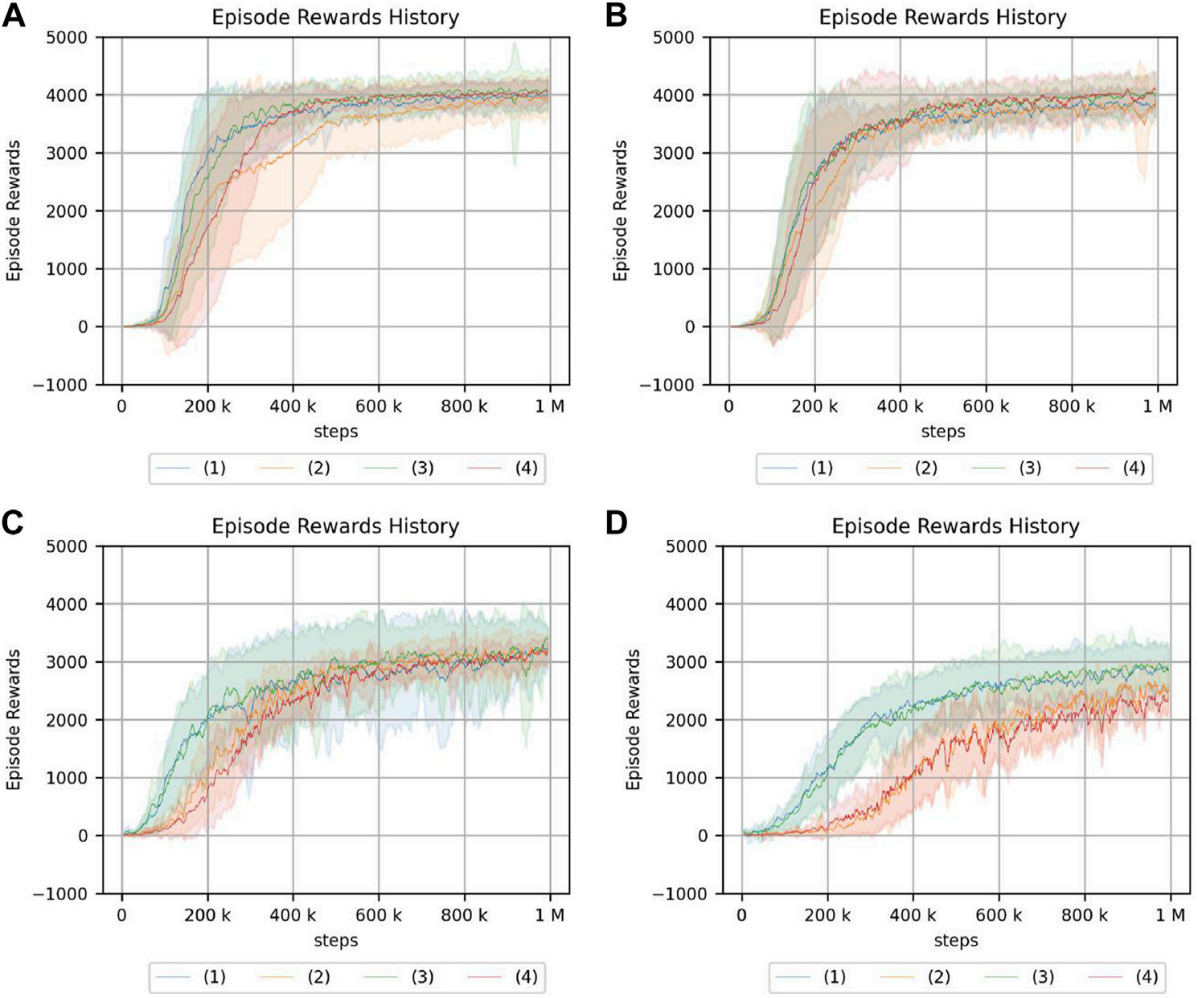
Return of the crank rotation. **(A)** Continuum robot arm: no obstacle, no randomness of initial posture. **(B)** Continuum robot arm: no obstacle, high randomness of initial posture. **(C)** 7-DoF arm: no obstacle, no randomness of initial posture. **(D)** 7-DoF arm: medium obstacle size, and high randomness of initial posture. The legend indicates the position of the crank and initial angle of the handle: (1) random/fixed, (2) random/random, (3) fixed/fixed, (4) fixed/random.

**FIGURE 9:**
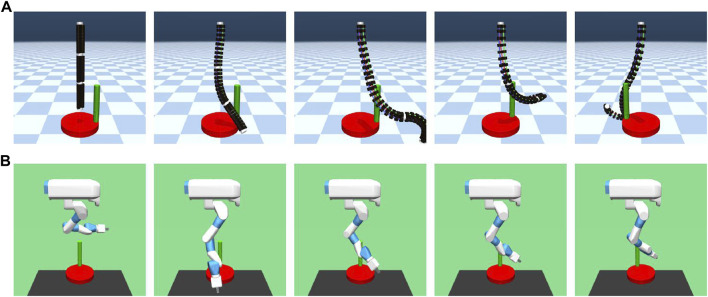
Performing a crank rotation task according to an acquired policy. **(A)** continuum robot arm. **(B)** 7-DoF arm.

The experimental results in the crank rotation environment are shown in Figure 8. Here, only the representative results are shown. The results for all experimental conditions are provided in the Supplementary Material. Figure 9 also illustrates the task being carried out by the acquired policy.

From the experimental results of the continuum robot arm in an obstacle-free environment, the cumulative reward shows that crank rotation depends on the randomness of neither the environment nor initial posture. However, for the 7-DoF arm robot, the cumulative reward does depend on the randomness of the environment, which is the initial handle rotation angle. Moreover, the speed of learning verifiably slows down because of such randomness. This tendency is also observed in the environment with obstacles, but the dependency on the initial handle rotation angle of the 7-DoF arm robot is more pronounced in the case with obstacles, and the difference is larger. Additionally, the reinforcement-learning results generally deteriorate if the obstacle is large.

The reason the performance of the 7-DoF arm robot deteriorates in terms of the cumulative reward for reinforcement learning even in the case of a small obstacle may be that the crank cannot be turned properly because of the constraints of the robot configuration. In fact, the robot appears to flick the handle if there is an obstacle present. In addition, the performance drops significantly if there is a large obstacle and the initial angle of the handle is random. This is because it is difficult to turn the crank if the handle is not in a place where it could be played well, whereas the popping motion is acquired.

From the experimental results in an obstacle-free environment, the continuum robot arm is more robust to environmental randomness and less robust to the initial posture of the robot. In contrast to the continuum robot arm, the 7-DoF arm robot is less robust to environmental randomness considering the position of the handle but more robust to the initial posture of the robot. The effect of the randomness in the position of the handle is the same as in the case of an obstacle. The fact that the speed of convergence of reinforcement learning slows down if there is randomness in the initial angle of the handle for the 7-DoF arm robot may be because it requires more precise control than the continuum robot arm for tasks that require contact with objects. However, the continuum robot arm may have been able to perform the task even if it moved slightly imprecisely in tasks where it only needed to get a feel for the environment by interacting with it.

For the 7-DoF arm robot, the cumulative reward is smaller in the presence of small obstacles, and this significantly impacts the learning performance. Therefore, the continuum robot arm is proposed to be more suitable for position control in the presence of small obstacles. However, if small obstacles are present, the cumulative reward of reinforcement learning for the continuum robot arm decreases significantly, whereas the effect on the 7-DoF arm robot is smaller than that of the continuum robot arm. This is because the 7-DoF arm robot successfully rotates around large obstacles, while the continuum robot arm cannot. Moreover, if the obstacle is large, the continuum robot arm’s “inability to perform fine motion control” outweighs its “ability to interact with the environment using its hands”.

#### 5.2.2 Results and discussion of peg-in-hole

**FIGURE 10:**
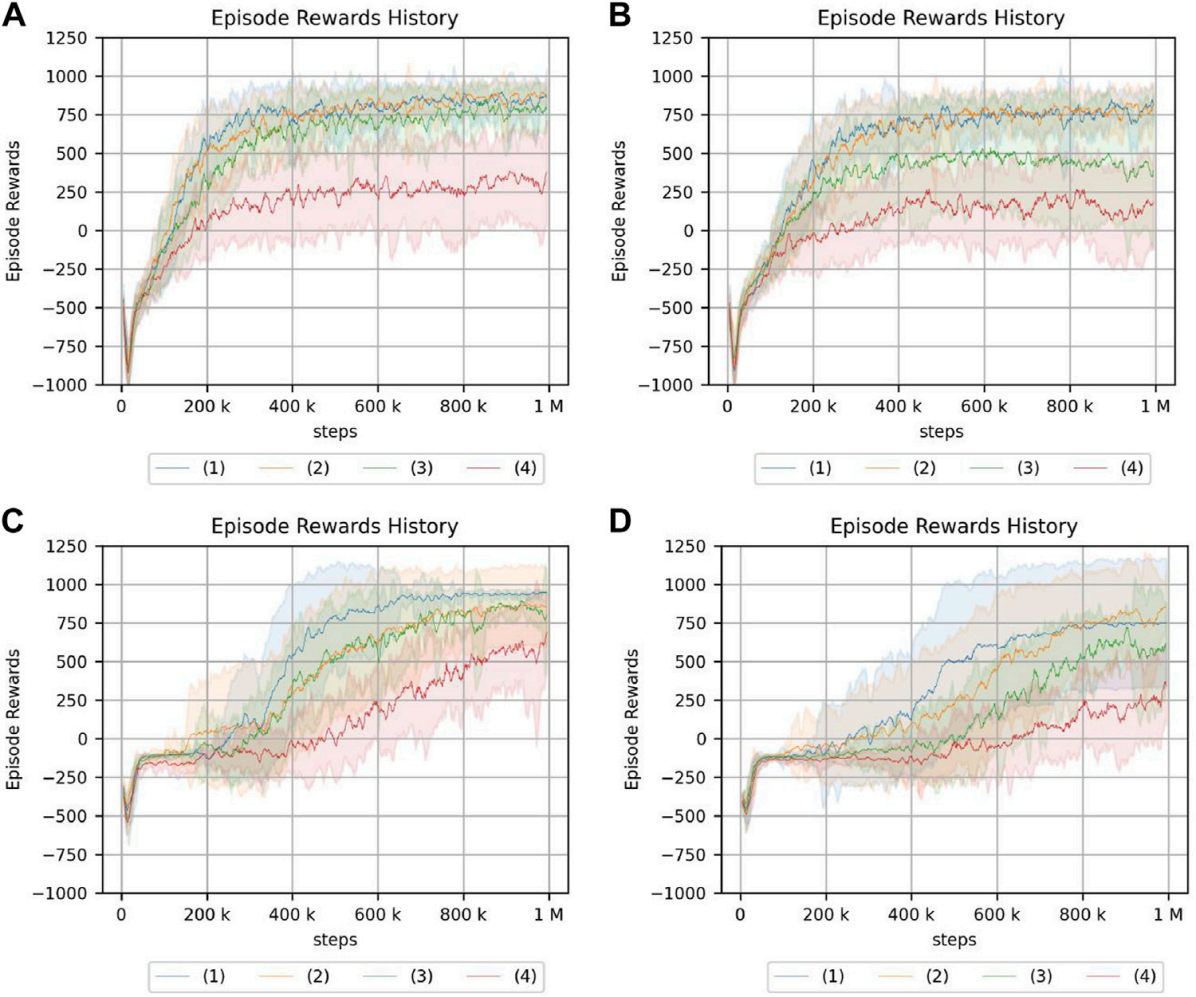
Return of peg-in-hole. **(A)** Continuum robot arm: no obstacle, no randomness of initial posture. **(B)** Continuum robot arm: no obstacle, high randomness of initial posture. **(C)** 7-DoF arm: no obstacle, no randomness of initial posture. **(D)** 7-DoF arm: medium obstacle size, and high randomness of initial posture. Randomness of a hole position: (1) nil, (2): low, (3): medium, (4): high.

Next, the experimental results in the peg-in-hole environment are shown in Figure 10. Here, only the representative results are shown. The results for all experimental conditions are given in the Supplementary Material. All experiments were terminated with the same number of samples. This is because, in this study, it was sufficient to know the trend in each environment. Therefore, it is not necessary to compare the final performance of the two environments, and the comparison of learning speeds is not needed.

From the experimental results in an obstacle-free environment, the continuum robot arm is observed to be considerably affected by the randomness of the environment. The randomness of the initial state is also found to be more susceptible if the randomness of the hole position is high. Furthermore, if the randomness of the initial posture increases, the tolerance to the randomness of the hole position decreases. For the continuum robot arm, if the randomness of the initial posture is high, the effect of the randomness in the hole position for peg-in-hole is dominant, regardless of obstacles.

For the 7-DoF arm robot, the effects of initial posture and hole location randomness are evidently negligible. In the case of the 7-DoF arm robot, the cumulative reward is lower if there is an obstacle, especially if there is no randomness in the initial posture, indicating that the effect of the obstacle cannot be ignored.

From the experiments in the environment without obstacles, the cumulative reward evidently decreases if the initial posture of the continuum robot arm is disorderly, unlike the crank rotation environment. However, the ability to respond to obstacles is the same as in the crank rotation environment.

In addition, the peg-in-hole task requires precise position control and compliance control. The highest cumulative reward is obtained if there is no environmental randomness in the 7-DoF arm robot. Moreover, the equivalent cumulative reward is obtained if there is no environmental randomness, indicating that the rigid robot has an advantage over the soft robot in precise control. However, if the environment is random, there are cases in which the continuum robot arm has a better cumulative reward, indicating that the soft robot may be more suitable.

For peg-in-hole, reinforcement learning is achieved in the presence of large obstacles although the performance of the continuum robot arm is degraded because of the large passive degree of freedom. This contrasts the case of 7-DoF arm robot, which does not insert the stick into the hole at all. These results indicate that passive degrees of freedom and redundancy are effective for some tasks.

### 5.3 Results and discussion of ball throwing

**FIGURE 11:**
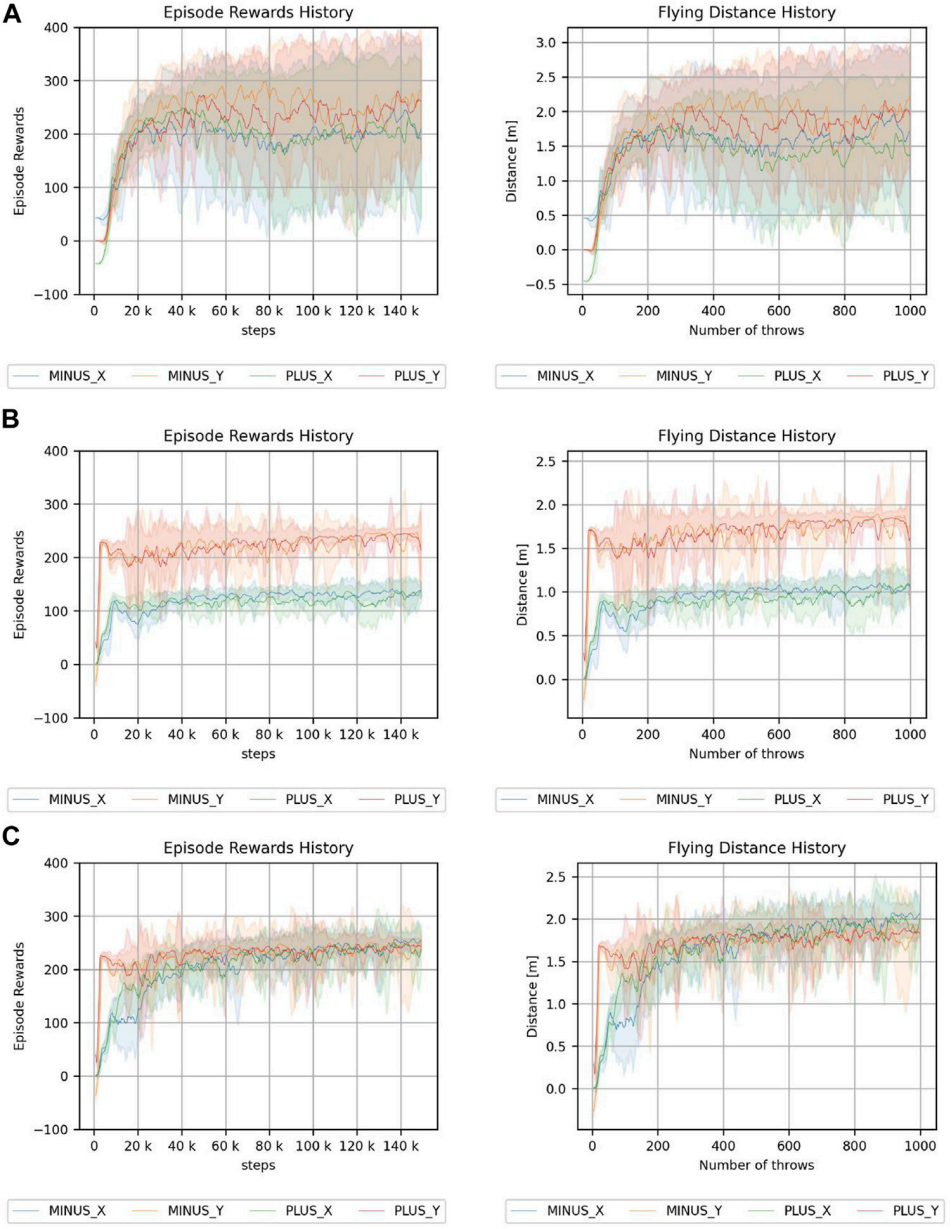
Results of throwing a ball experiment. **(A)** Continuum robot arm. **(B)** 7-DoF arm robot. **(C)** 7-DoF arm robot using a root joint. The legend indicates the direction of the target.

The results of the throwing experiment are shown in Figure 11. The results show the cumulative reward and distance, i.e., the distance of the target direction vector from the initial ball position to the landing point. For the continuum robot arm, the results are almost the same, regardless of the direction; however, for the 7-DoF arm robot, there is a difference depending on the direction. In the environment where the root does not rotate, the difference is larger than that in the environment where the root can rotate, possibly because of fewer degrees of freedom.

These results indicate that the continuum robot arm can achieve similar performance for cumulative reward, regardless of the direction; however, the 7-DoF arm robot is highly dependent on the direction. Although the 7-DoF arm robot has redundancy, some motions are observed to be easier than others, depending on the joint arrangement, and the difference cannot be completely prevented by rotating the root. Although this aspect can be improved by statically rotating the arm beforehand, the anisotropy may become a problem in end-to-end learning. However, the continuum robot arm is less dependent on the direction because of the symmetry of the structure and is consequently more suitable for anisotropic tasks.

### 5.4 Discussion on characteristic analysis of continuum arm using reinforcement learning

The properties of the typical hanging type continuum robot arm used in this study are discussed through a comparison with a rigid 7-DoF robot manipulator.

The results of the throwing experiments indicate that the continuum robot arm is more resistant to anisotropy than the 7-DoF robot arm. However, this may be because of the symmetrical structure of the robot used in this study. Additionally, such a structure is easy to realize in the continuum robot arm.

From the experimental results in the crank rotation and peg-in-hole environments, the rigid 7-DoF robot manipulator performed noticeably well in the latter environment where precise position control is required. However, in the crank rotation environment, where some choice of action is allowed and the aim is to rotate the crank, the continuum robot arm performed particularly well if environmental randomness exists for cumulative reward. This may be because the crank rotation task can be performed by only applying force to the crank in a specific direction, and does not require precise control while the direction of force application is correct. For continuum robot arm, the elasticity and continuity of the body makes it easy for it to wrap around the handle and push it, as well as absorb the randomness of the environment. Furthermore, since the reward for turning the crank in this experiment is angular velocity and not rotation angle, it is possible that the initial environmental randomness does not affect the reward.

However, in the peg-in-hole environment, where position control is required, the initial randomness negatively influences learning. For the same reason, it is possible that the randomness of the initial position and position of the hole in the peg-in-hole environment significantly impact learning by considerably affecting the task execution. However, the fact that this effect is particularly significant for the continuum robot arm suggests that it may be difficult to obtain a wide range of precise motions by simple reinforcement learning.

The initial posture of the robot, obtained by the given clutter, differs from that of a 7-DOF arm in that a continuum robot arm contains many poses that cannot be reached by actuator manipulation. This is due to the softness and redundancy of the robot.

As for the initial state randomness, in real world, the continuum robot arm itself may shake because of wind, the task completed prior, or being lifted by human hands. We believe these properties are responsible for the randomness of the environment affecting the experimental results of the continuum robot arm.

Reducing these effects may help facilitate learning; a simple improvement method is proposed in the next section as well.

Referring to the characteristics in Table 1, we consider that the continuum robot arm is superior to the 7-DoF arm in redundancy and anisotropy. The results indicate that the 7-DoF arm is highly effective for static precision control. On the other hand, the results indicate that the continuum robot arm is highly effective for dynamic movements, as demonstrated by the ball-throwing task. The results differ depending on the task set for the randomness of the initial state and the environment.

## 6 Proposed reinforcement learning method based on results of characteristic analysis

Based on the experimental results of characterizing a continuum robot arm, the disadvantage of continuum robot arm control is the unavoidable effect of initial state randomness in an environment where precise position control is required. Considering the results, a method is proposed to improve the performance of reinforcement learning in a continuum robot arm.

### 6.1 Methods

In this section, a simple method is proposed to reduce the effect of the initial posture randomness on the continuum robot arm by adding a certain period of time for the arm to be in a contracted state before the start of the episode. In this study, a type of continuum robot arm that can be extended by applying force to the actuators was used. Therefore, the command values of all nine actuators were set to zero to reduce the randomness in the initial posture by allowing a certain period of time without any extension. In this experiment, this mechanism is called the “reset phase.”

Continuum robot arm robots are susceptible to their own initial state. Therefore, a reset phase to fix the initial state is considered adequate. However, this reset phase resets its own state and does not affect the randomness of the surrounding environment, such as the target position.

The time to give the zero-command value was 500 steps in all experiments, and because each time-step was 40 ms in all environments, the time was 20 s.

SAC was employed as the reinforcement learning algorithm, same as in the characteristic analysis. The rest of the settings were also the same. Any reinforcement learning method can be employed and does not have to be reinforcement learning because of the characteristics of the operation.

In this section, the peg-in-hole and crank rotation environments were used without any obstacle using a continuum robot arm. Additionally, the effects of initial posture randomness were considered, same as in the characteristic analysis.

### 6.2 Results

**FIGURE 12:**
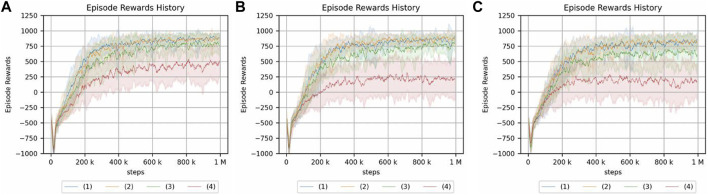
Peg-in-hole using a reset phase for a continuum arm. Each of the three graphs has a different magnitude of noise in the initial posture. **(A)** No randomness. **(B)** Low randomness. **(C)** High randomness. Randomness of a hole position: (1) nil, (2) low, (3) medium, and (4) high.

**FIGURE 13:**
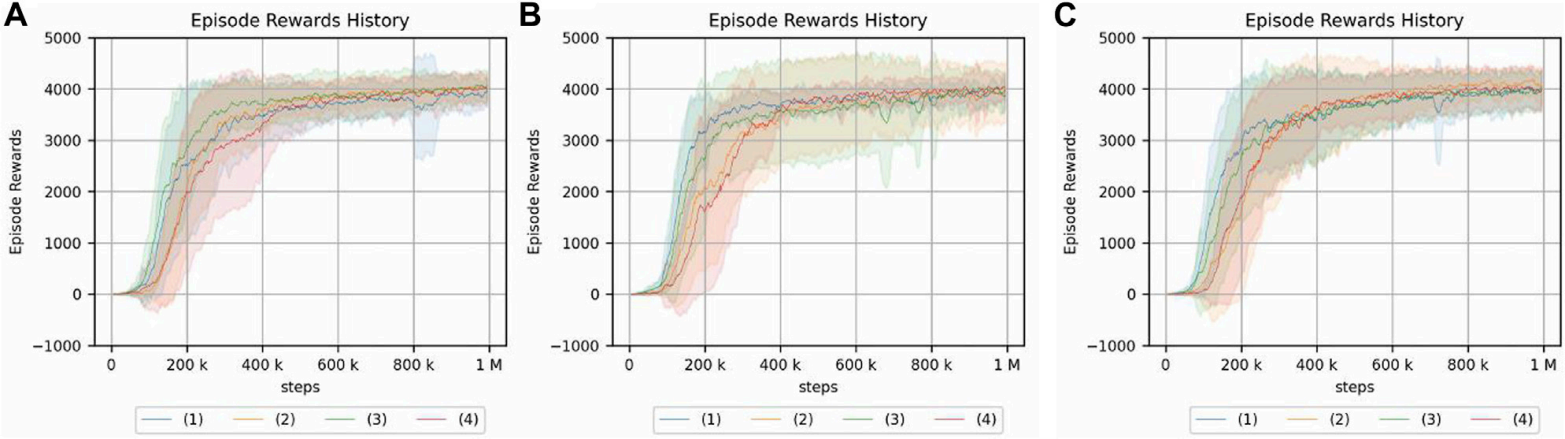
Crank rotation using a reset phase for a continuum arm. Each of the three graphs has a different magnitude of noise in the initial posture. **(A)** No randomness of initial posture. **(B)** Low randomness of initial posture. **(C)** High randomness of initial posture. The legend indicates the position of the crank and initial angle of the handle: (1) random/fixed, (2) random/random, (3) fixed/fixed, and (4) fixed/random.

The results of the experiment are shown in Figures 12, 13. Note that the same type of experiment was performed 16 times, each with different seed values.

For the peg-in-hole environment, there is no difference in the cumulative reward if there is no randomness in the initial posture. Moreover, the randomness in the environment, i.e., the location of the hole, is low compared to Figure 10, which is the result of an experiment without a reset phase. However, if the randomness of the hole position is high in the absence of initial randomness or if the randomness of the hole position is nil, low, or medium in the presence of initial randomness, the reset phase improves the cumulative reward obtained by reinforcement learning. In the same case, if the randomness of the hole locations is particularly high, the reset phase does not contribute to increasing the cumulative reward.

As for the crank rotation environment, the results without a reset phase (Figure 8) show limited effect of initial posture randomness, and the same is true for the present results.

**FIGURE 14:**
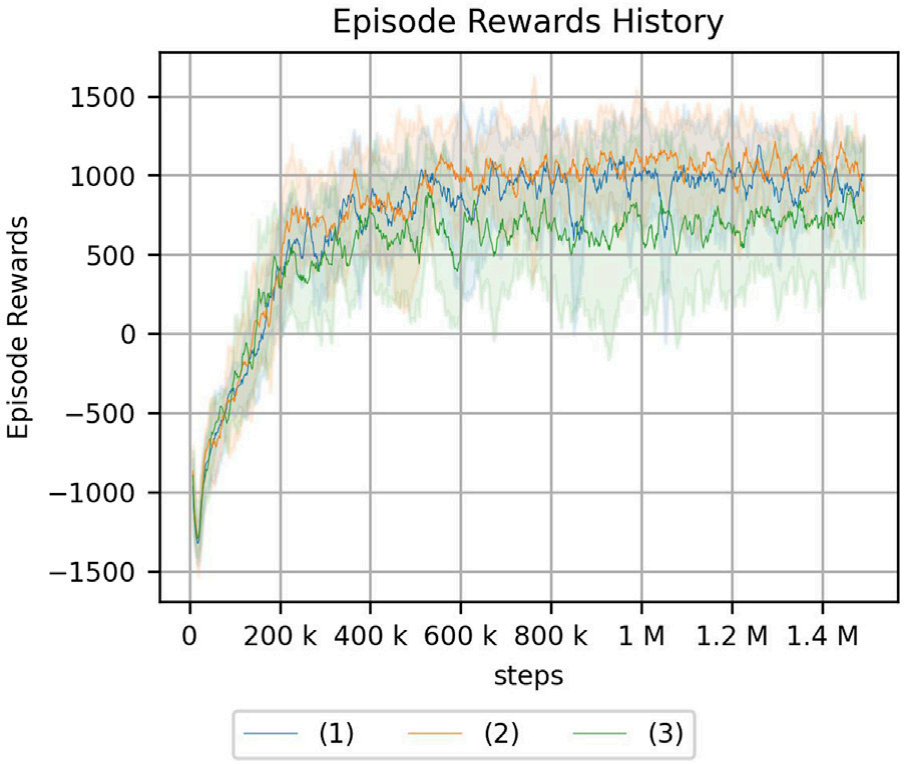
Result of peg-in-hole task with other conditions using a continuum arm. The number of steps per epoch: 1500 (the number of episodes: 1000). The legend indicates the randomness of initial posture: (1) nil, (2) low, and (3) high.

In addition, the actual number of steps required for learning in the simulation is 500 steps more per episode in the proposed method, and we cannot deny the possibility that this is because of the increase in the number of samples. An experiment with 1500 steps per episode was conducted to confirm that the cumulative reward obtained as a result of reinforcement learning is not larger because of an increase in the number of samples. The corresponding result is shown in Figure 14. This result clearly indicates that the learning performance does not improve because of the increase in the number of samples.

### 6.3 Discussion

The proposed method aims to reduce the effect of initial posture randomness, which is useful for the peg-in-hole environment, but not for the crank-rotation environment. As mentioned earlier, peg-in-hole requires precise position control to insert a peg into a hole, whereas crank rotation requires only pushing a handle and tends to require large motions. Therefore, the control required for crank rotation is less precise than that for peg-in-hole in which the robot uses the contact with the handle to touch it. Consequently, even if the initial posture is random, the performance may be sufficiently good for the cumulative reward of reinforcement learning. It is also possible that the large amount of motion was absorbed by the system. However, for peg-in-hole, precise position control is required to reach the approximate position of the hole. In the absence of a reset phase, the effect of the initial posture may cause a decrease in the cumulative reward of reinforcement learning. These results indicate that the continuum robot arm, which is one of the soft robots, is not as good at precise position control as the 7-DoF arm robot, which is a stiffer robot; however, eliminating the effect of the initial posture randomness can improve the performance to a certain extent.

This approach could also be helpful when applying control models learned from simulations on soft robots to real robots. It has the potential to improve robustness to disturbances such as load and deviations between the simulation model and the real robot.

## 7 Conclusion

In this study, for the first time, multiple types of tasks were performed by a single continuum robot arm in a simulation. The performance of the continuum robot arm was comprehensively examined for the first time by comparison with a rigid robot. Furthermore, based on the results of this investigation, a method for improving performance in reinforcement learning was proposed.

We first verified if the simulation model created can substitute for experiments on a real robot. Subsequently, the characteristics of a continuum robot arm, a soft robot, were analyzed by performing reinforcement learning on several tasks and comparing it with a rigid 7-DoF robot manipulator made of a rigid material in the simulation. Based on the results, a reset phase was incorporated as a reinforcement learning method for the continuum robot arm to improve its performance in tasks that require precise control. Notably that the continuum robot arm used in this study is one of the most common suspended continuum robot arms compared to other robots driven by actuators; therefore, it is an appropriate choice to study the general properties of continuum robot arms.

To analyze the characteristics of continuum robot arms, four tasks were performed using model-free reinforcement learning: reaching, crank rotation, peg-in-hole, and throwing. The comparison between the two types of robots demonstrated the effects of environmental randomness as well as the randomness of the robot’s initial posture, anisotropy, and behavior if there are obstacles in space. The randomness of the initial posture and presence of obstacles was observed to significantly impact the reinforcement learning for the continuum robot arm. To the best of our knowledge, no other study has investigated the characteristics of reinforcement learning and continuum robot arms in such a comprehensive manner. Moreover, no studies have been found in which several different tasks were performed using the same continuum robot arm through reinforcement learning, as in this study.

The proposed reinforcement learning method that incorporates a reset phase is particularly useful for tasks that require precise control, and may be useful for controlling a continuum robot arm that is not good at such tasks.

As for future work, although we performed reinforcement learning of the continuum robot arm using multiple tasks, the number of tasks is limited; therefore, verifying the results of this study using a wide range of tasks is necessary. In addition, the characteristics of the soft robot are not limited to those discussed in this study; for example, viscoelasticity, the degree of redundancy, and differences due to the drive source are some other aspects. Therefore, further clarifying the characteristics of the continuum robot arm and proposing a new reinforcement learning method may be achievable by considering another characteristic. In addition, the continuum robot arm is more suitable for tasks involving interaction with the environment than the rigid 7-DoF robot manipulator; however, the effects of obstacles on the continuum robot arm cannot be ignored. Therefore, it is necessary to develop a method that can both reduce the influence of obstacles and maintain tolerance to environmental randomness.

## Data Availability

The raw data supporting the conclusion of this article will be made available by the authors, without undue reservation.
