# Supplementary material for: Characterization of continuum robot arms under reinforcement learning and derived improvements
Source: Front Robot AI. 2022 Sep 1;9:895388. doi: 10.3389/frobt.2022.895388 (PMC9475256; doi:10.3389/frobt.2022.895388)
Supplement: Supplementary file 1 [file DataSheet1.PDF]

Supplementary Material

1 ALL RESULTS FOR CHARACTERISTIC ANALYSIS OF CONTINUUM ROBOT ARMS USING REINFORCEMENT LEARNING

In this section, all experimental results for the characterization of the continuum robot arm are presented.

1.1 Crank Rotation

As regards the experimental results of the crank rotation task in an obstacle-free environment, the results for the continuum arm robot are shown in figure S1, and those for the 7-DoF arm robot are presented in figure S2.

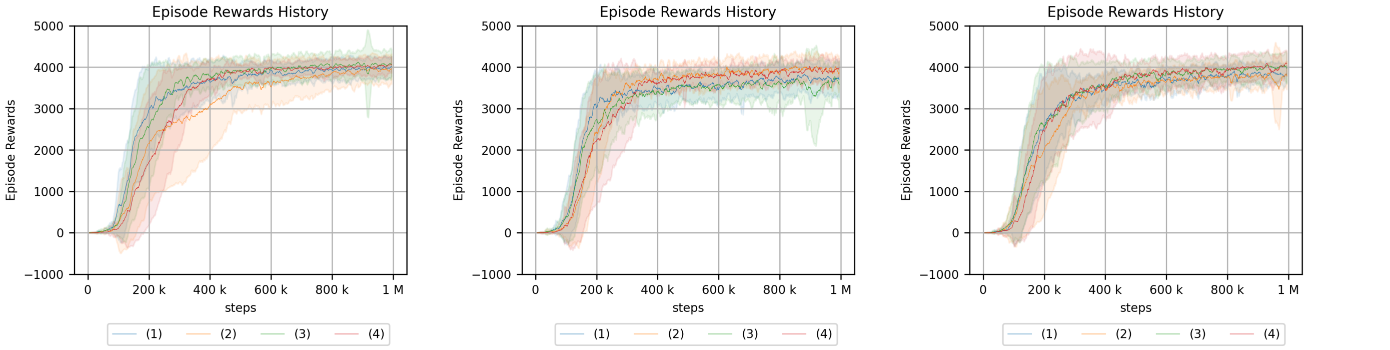

(a) Randomness of initial posture: nil. (b) Randomness of initial posture: low. (c) Randomness of initial posture: high.

Figure S1. Return of the crank rotation experiment (continuum arm). Each of the three graphs corresponds to a different magnitude of noise in the initial posture. The legend indicates the position of the crank and initial angle of the handle: (1) random/fixed, (2) random/random (3) fixed/fixed, and (4) fixed/random.

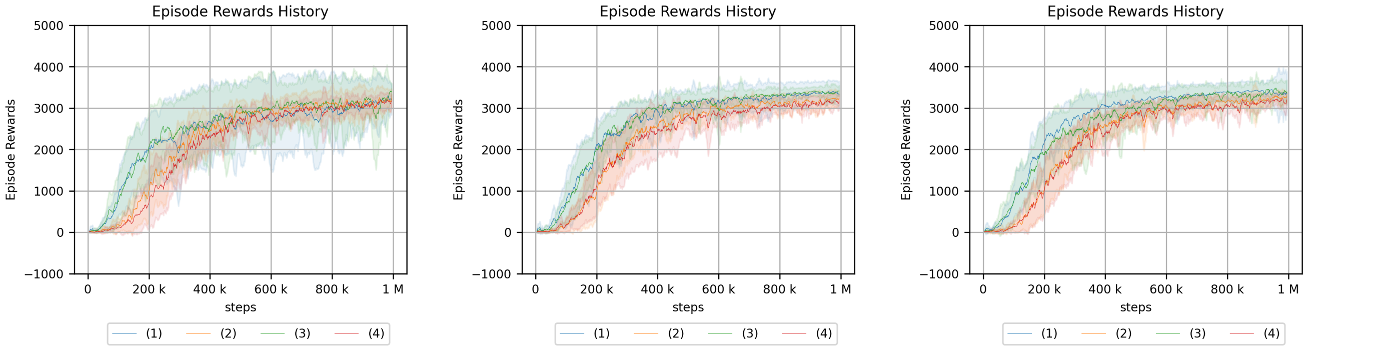

(a) Randomness of initial posture: nil. (b) Randomness of initial posture: low. (c) Randomness of initial posture: large.

Figure S2. Return of the crank rotation experiment (7-DoF arm). Each of the three graphs corresponds to a different magnitude of noise in the initial posture. The legend indicates the position of the crank and initial angle of the handle: (1) random/fixed, (2) random/random (3) fixed/fixed, and (4) fixed/random.

Experimental results in the presence of large, medium-sized, and small obstacles in the environment of crank rotation with a continuum arm robot are shown in figures S3, S4, and S5. As regards the a 7-DoF arm, figures S6, S7, and S8 depict the results for no, low, and high initial posture randomness, respectively.

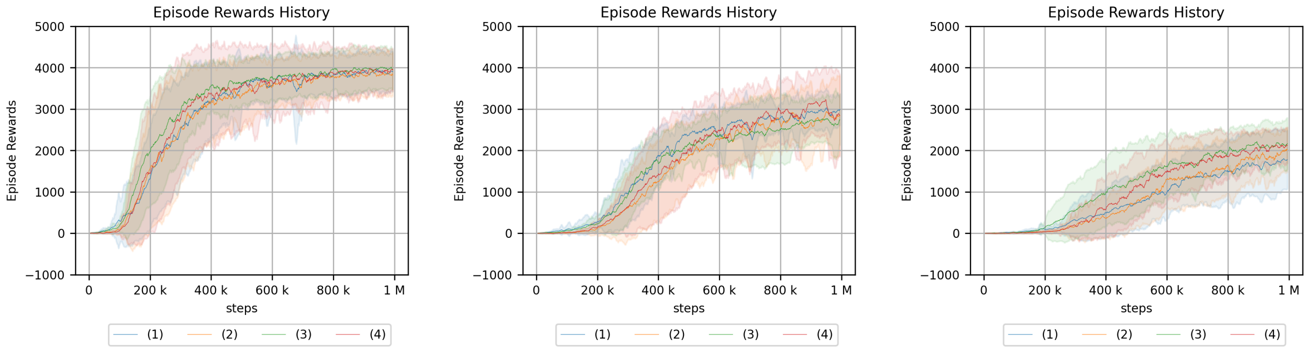

(a) Obstacle size: small. (b) Obstacle size: medium. (c) Obstacle size: large.

Figure S3. Return of the crank rotation with an obstacle experiment (continuum arm). Randomness of initial posture is nil. The legend indicates the position of the crank and initial angle of the handle: (1) random/fixed, (2) random/random, (3) fixed/fixed, and (4) fixed/random.

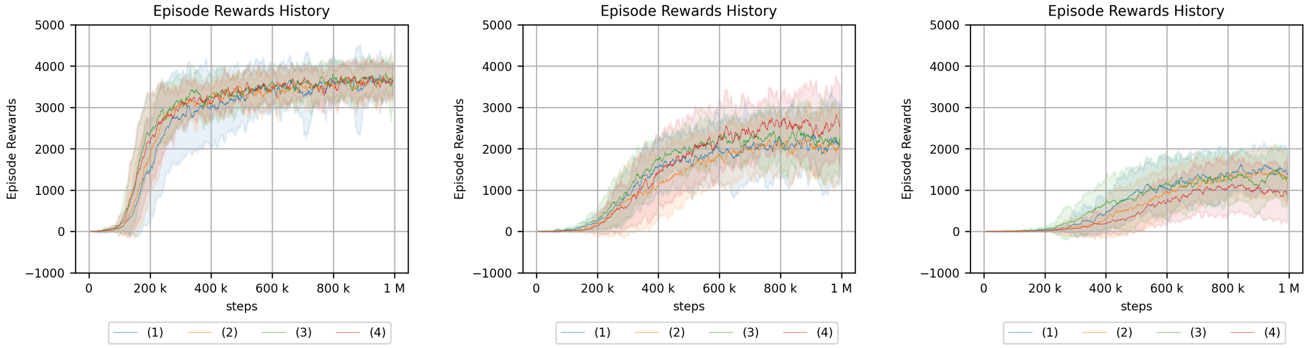

(a) Obstacle size: small. (b) Obstacle size: medium. (c) Obstacle size: large.

Figure S4. Return of the crank rotation with an obstacle experiment (continuum arm). Randomness of initial posture is low. The legend indicates the position of the crank and initial angle of the handle: (1) random/fixed, (2) random/random, (3) fixed/fixed, and (4) fixed/random.

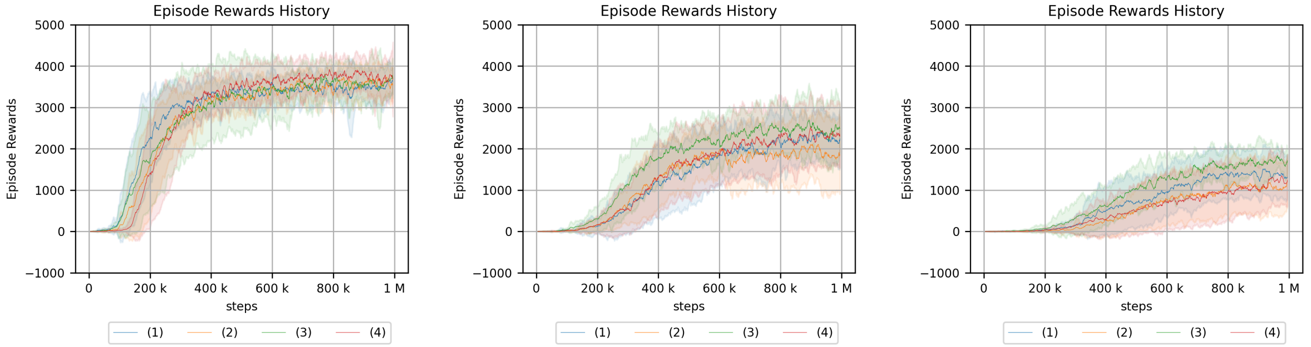

(a) Obstacle size: small. (b) Obstacle size: medium. (c) Obstacle size: large.

Figure S5. Return of the crank rotation with an obstacle experiment (continuum arm). Randomness of initial posture is high. The legend indicates the position of the crank and initial angle of the handle: (1) random/fixed, (2) random/random, (3) fixed/fixed, and (4) fixed/random.

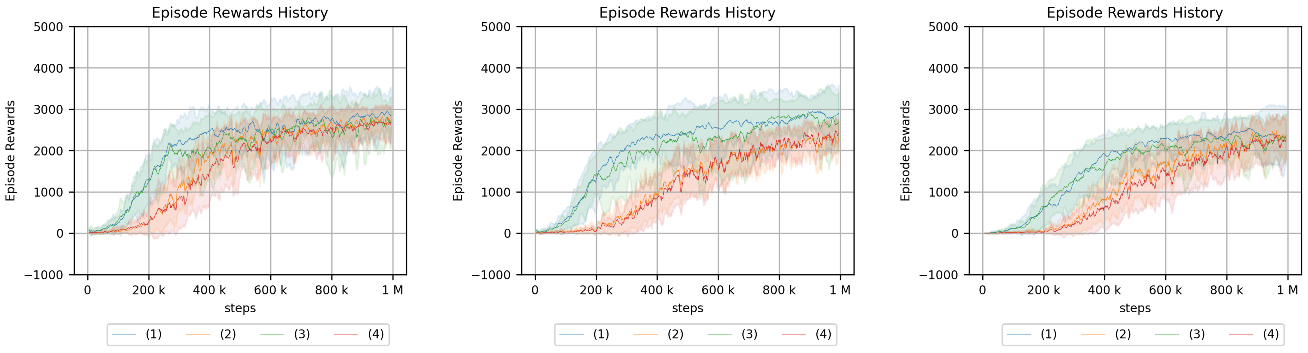

(a) Obstacle size: small. (b) Obstacle size: medium. (c) Obstacle size: large.

Figure S6. Return of the crank rotation with an obstacle experiment (7-DoF arm). Randomness of initial posture is nil. The legend indicates the position of the crank and initial angle of the handle: (1) random/fixed, (2) random/random, (3) fixed/fixed, and (4) fixed/random.

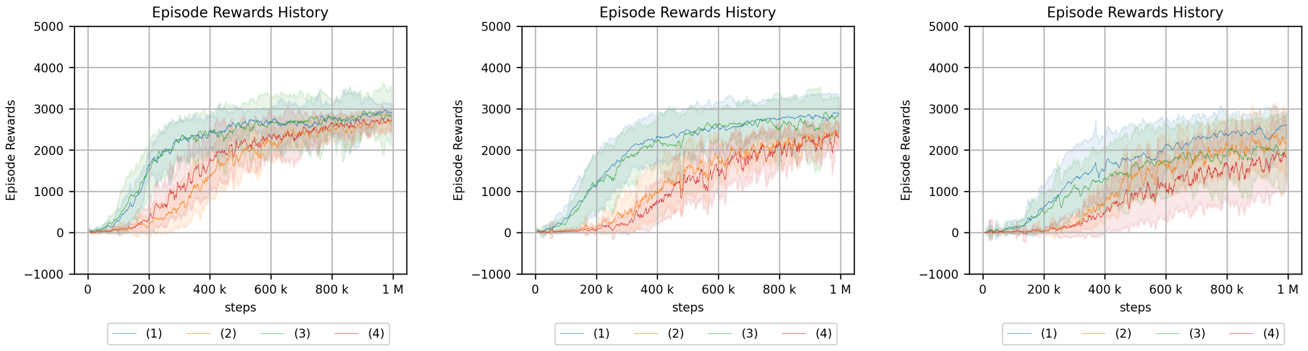

(a) Obstacle size: small. (b) Obstacle size: medium. (c) Obstacle size: large.

Figure S7. Return of the crank rotation with an obstacle experiment (7-DoF arm). Randomness of initial posture is low. The legend indicates the position of the crank and initial angle of the handle: (1) random/fixed, (2) random/random, (3) fixed/fixed, and (4) fixed/random.

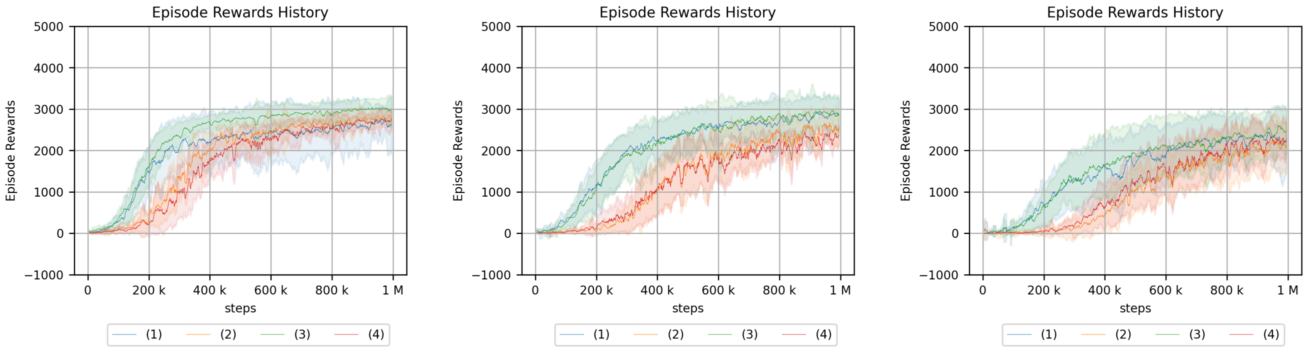

(a) Obstacle size: small. (b) Obstacle size: medium. (c) Obstacle size: large.

Figure S8. Return of the crank rotation with an obstacle experiment (7-DoF arm). Randomness of initial posture is high. The legend indicates the position of the crank and initial angle of the handle: (1) random/fixed, (2) random/random, (3) fixed/fixed, and (4) fixed/random.

1.2 Peg Insertion

For the experimental results of the peg-in-hole task in an obstacle-free environment, the results for the continuum arm robot are shown in figure S9, and the results for the 7-DoF arm robot are shown in figure S10.

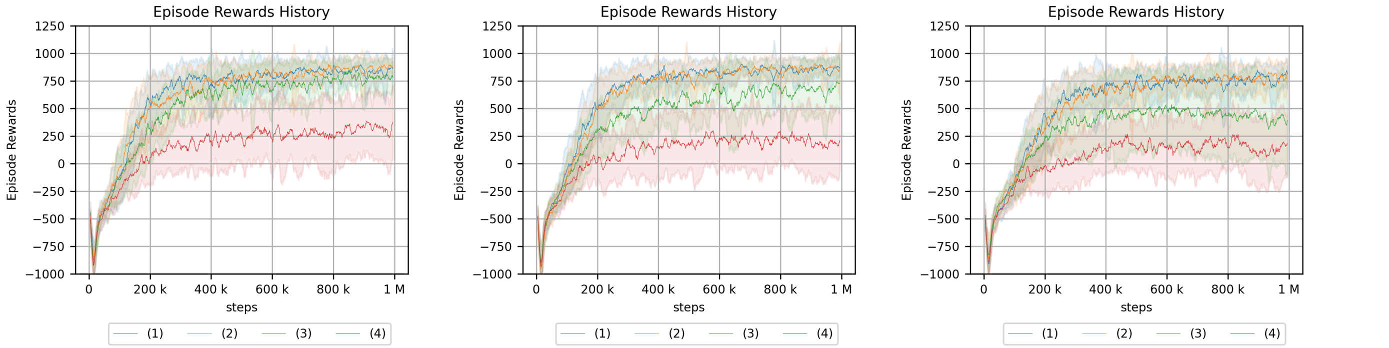

(a) Randomness of initial posture: nil. (b) Randomness of initial posture: low. (c) Randomness of initial posture: large.

Figure S9. Return of the peg-in-hole experiment (continuum arm). Each of the three graphs has a different magnitude of noise in the initial posture. Randomness of a hole position: (1) nil, (2) low, (3) medium, and (4) high.

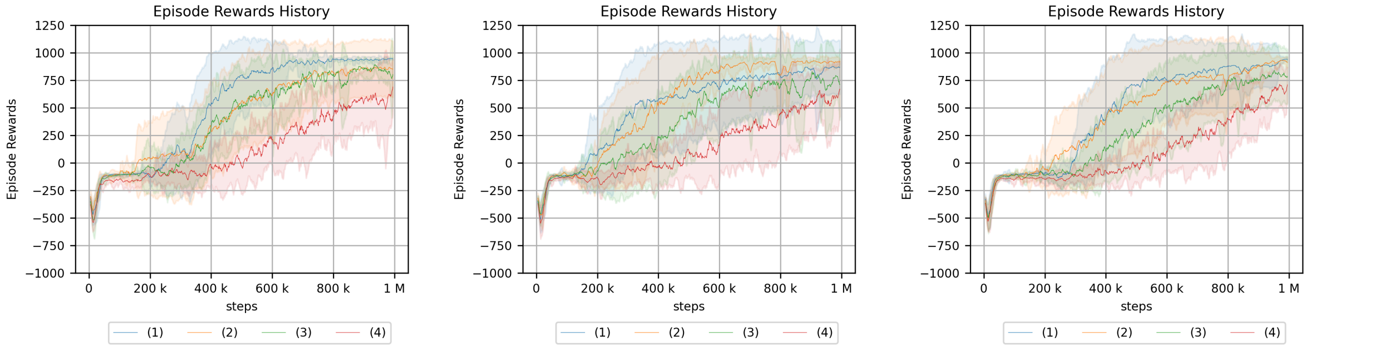

(a) Randomness of initial posture: nil. (b) Randomness of initial posture: low. (c) Randomness of initial posture: large.

Figure S10. Return of the peg-in-hole experiment (7-DoF arm). Each of the three graphs has a different magnitude of noise in the initial posture. Randomness of a hole position: (1) nil, (2) low, (3) medium, and (4) high.

Experimental results in the presence of large, medium-sized, and small obstacles in the peg-in-hole assembly environment with a continuum arm robot are shown in figures S11, S12, and S13. Similarly, results for the 7-DoF arm are shown in figures S14, S15, and S16 no, small, and high initial posture randomness, respectively.

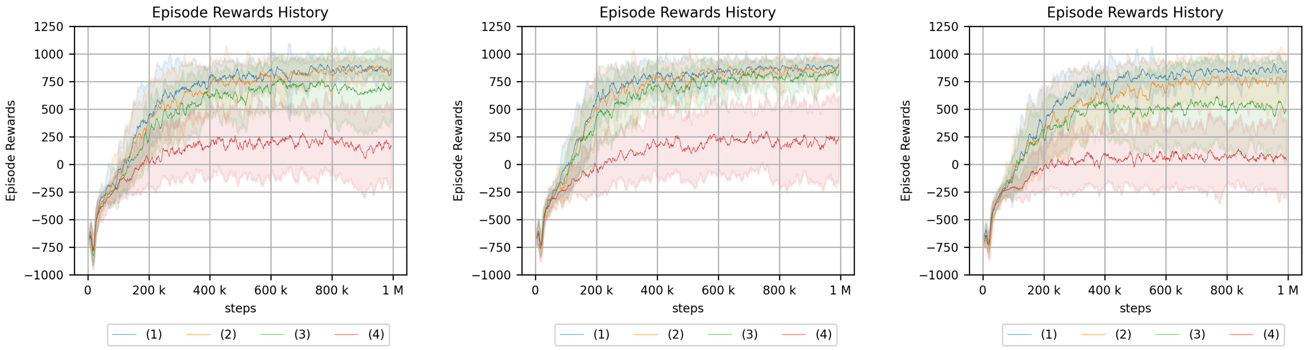

(a) Obstacle size: small. (b) Obstacle size: medium. (c) Obstacle size: large.

Figure S11. Return of the peg-in-hole assembly with an obstacle experiment (continuum arm). Randomness of initial posture is nil. Randomness of a hole position: (1) nil, (2) low, (3) medium, and (4) high.

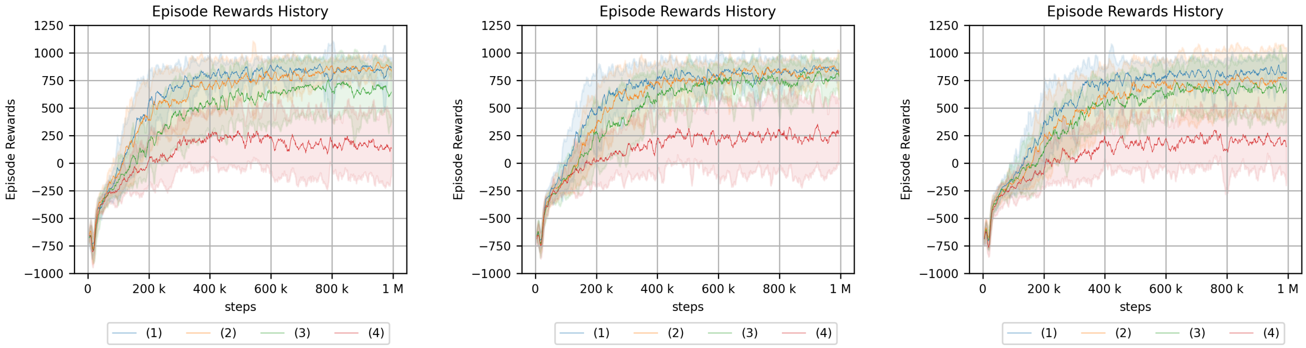

(a) Obstacle size: small. (b) Obstacle size: medium. (c) Obstacle size: large.

Figure S12. Return of the peg-in-hole assembly with an obstacle experiment (continuum arm). Randomness of initial posture is low. Randomness of a hole position: (1) nil, (2) low, (3) medium, and (4) high.

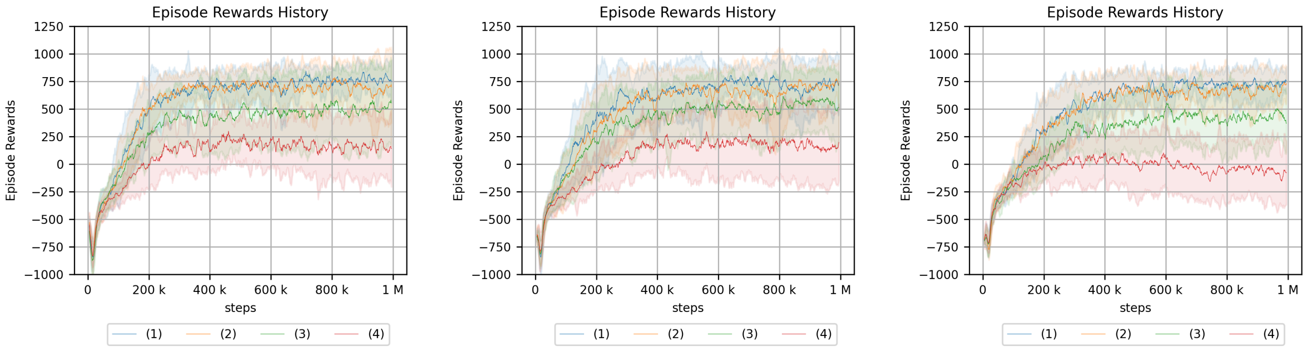

(a) Obstacle size: small. (b) Obstacle size: medium. (c) Obstacle size: large.

Figure S13. Return of the peg-in-hole assembly with an obstacle experiment (continuum arm). Randomness of initial posture is high. Randomness of a hole position: (1) nil, (2) low, (3) medium, and (4) high.

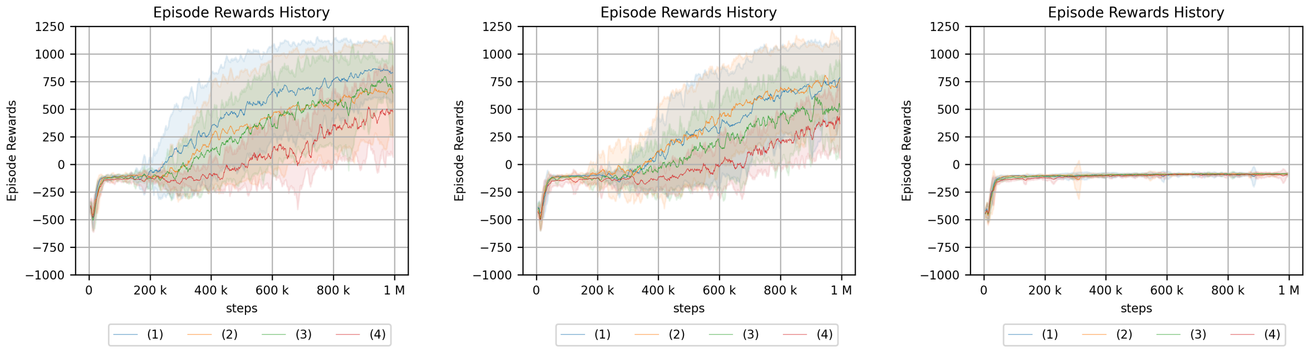

(a) Obstacle size: small. (b) Obstacle size: medium. (c) Obstacle size: large.

Figure S14. Return of the peg-in-hole assembly with an obstacle experiment (7-DoF arm). Randomness of initial posture is nil. Randomness of a hole position: (1) nil, (2) low, (3) medium, and (4) high.

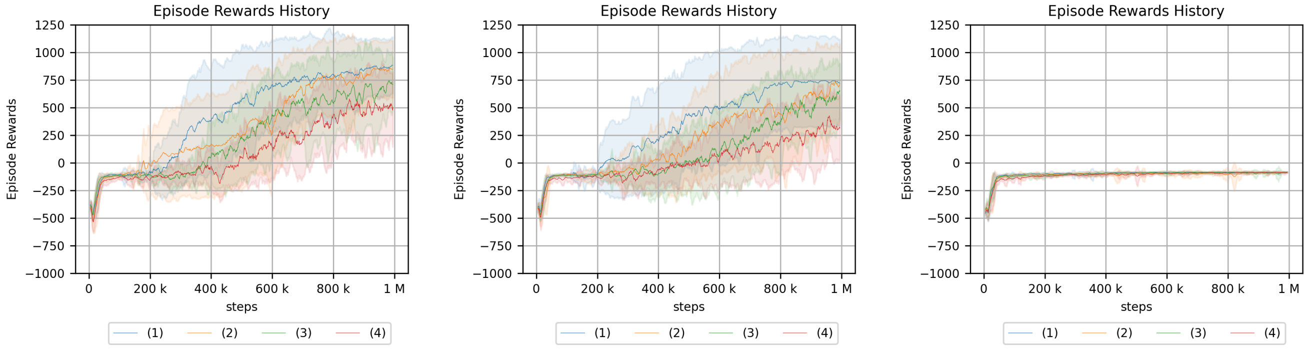

(a) Obstacle size: small. (b) Obstacle size: medium. (c) Obstacle size: large.

Figure S15. Return of the peg-in-hole assembly with an obstacle experiment (7-DoF arm). Randomness of initial posture is low. Randomness of a hole position: (1) nil, (2) low, (3) medium, and (4) high.

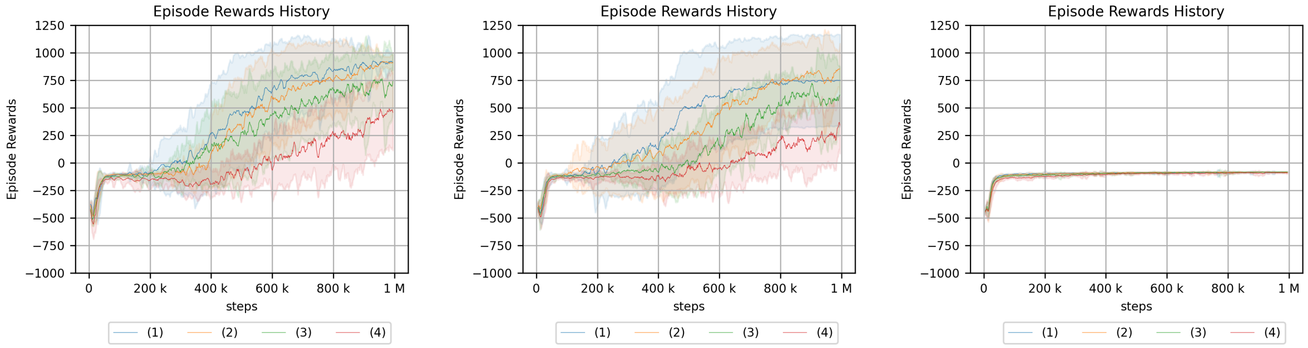

(a) Obstacle size: small. (b) Obstacle size: medium. (c) Obstacle size: large.

Figure S16. Return of the peg-in-hole assembly with an obstacle experiment (7-DoF arm). Randomness of initial posture is high. Randomness of a hole position: (1) nil, (2) low, (3) medium, and (4) high.

Figures S17 and S18 shows the task being performed using the learned policy if there are neither obstacles nor randomness in the learning process.

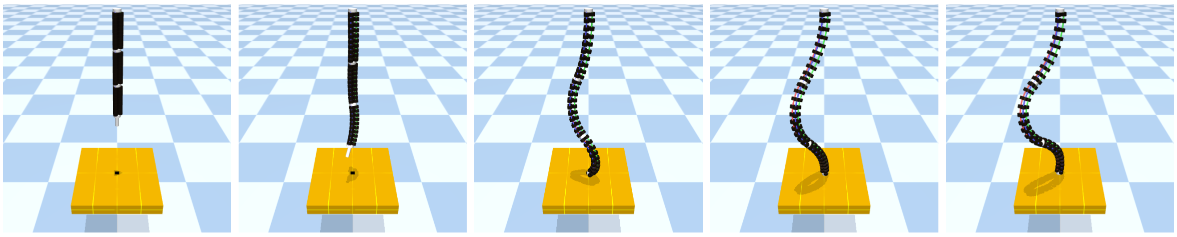

Figure S17. Performing a peg-in-hole task according to an acquired policy (continuum robot arm).

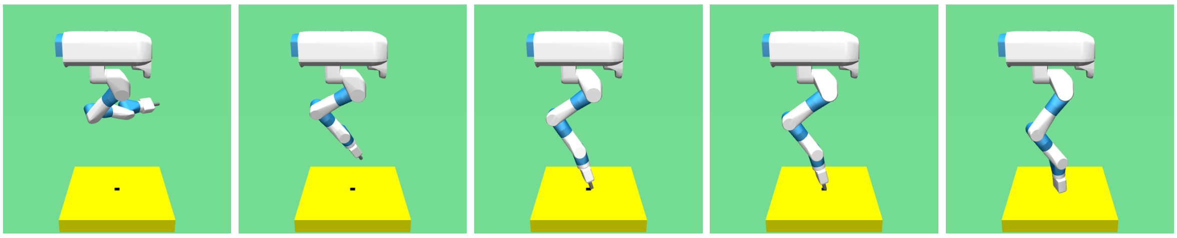

Figure S18. Performing a peg-in-hole rotation task according to an acquired policy (7-DoF arm).
